# Diversity and ecological function of urease-producing bacteria in the cultivation environment of *Gracilariopsis lemaneiformis*

**DOI:** 10.1007/s00248-023-02339-y

**Published:** 2024-01-23

**Authors:** Pengbing Pei, Muhammad Aslam, Hui Wang, Peilin Ye, Tangcheng Li, Honghao Liang, Qi Lin, Weizhou Chen, Hong Du

**Affiliations:** 1 Guangdong Provincial Key Laboratory of Marine Biotechnology, College of Science, Shantou University, Shantou, 515063 China; 2 Key Laboratory of Cultivation and High-Value Utilization of Marine Organisms in Fujian Province, Fisheries Research Institute of Fujian, Xiamen, 361000 China; 3 Guangdong Provincial Key Laboratory of Marine Disaster Prediction and Prevention, College of Science, Shantou University, Shantou, 515063 China; 4 Faculty of Marine Sciences, LUAWMS, Lasbela, 90150 Pakistan

**Keywords:** Urease-producing bacteria (UPB), Diversity, Ecological function, Urease, *Gracilariopsis lemaneiformis*

## Abstract

Urease-producing bacteria (UPB) provide inorganic nitrogen for primary producers by hydrolyzing urea, and play an important role in marine nitrogen cycle. However, there is still an incomplete understanding of UPB and their ecological functions in the cultivation environment of the red macroalgae *Gracilariopsis lemaneiformis*. This study comprehensively analyzed the diversity of culturable UPB and explored their effects on urea uptake by *G. lemaneiformis*. A total of 34 isolates belonging to four main bacterial phyla i.e. (Proteobacteria, Bacteroidetes, Firmicutes, and Actinobacteria) were identified through 16S rRNA sequencing and were screened for UPB by urea agar chromogenic medium assay and *ureC* gene cloning. Our data revealed that only 8 strains contained urease. All of these UPB exhibited different urease activities, which were determined by the Berthelot reaction colorimetry assay. Additionally, the UPB strain (G13) isolated from *G. lemaneiformis* with higher urease activity was selected for co-culture with *G. lemaneiformis* to explore its role in promoting or inhibiting nitrogen uptake by macroalgae. The results showed a significant increase in urea consumption in the culture medium and the total cellular nitrogen in *G. lemaneiformis* in the UPB-co culture group compared to the sterile group. This suggests that the selected UPB strain positively influences nitrogen uptake by *G. lemaneiformis.* Similarly, isotopic assays revealed that the δ^15^N content of *G. lemaneiformis* was significantly higher in the UPB-co culture than in the control group, where δ^15^N-urea was the only nitrogen source in the culture medium. This indicates that the UPB helped *G. lemaneiformis* to absorb more nitrogen from urea. Moreover, the highest content of δ^15^N was found in *G. lemaneiformis* with epiphytic bacteria compared to sterilized (i.e. control), showing that epiphytic bacteria, along with UPB, have a compound effect in helping *G. lemaneiformis* absorb more nitrogen from urea. Taken together, these results provide unique insight into the ecological role of UPB and suggest that urease from macroalgae environment-associated bacteria might be an important player in marine nitrogen cycling.

**Supplementary Information:**

The online version contains supplementary material available at 10.1007/s00248-023-02339-y.

## Introduction

Nitrogen (N) is not only an essential element for all life forms but also a pivotal macronutrient that limits biomass and primary productivity in the marine ecosystem [1, 2]. N compounds in the marine environment exist mainly in inorganic and organic forms [2, 3]. While algae and marine plants use inorganic N directly [4, 5], they can also utilize organic N sources when inorganic N is scarce [6]. Organic N includes proteins, amino acid/sugars, nucleic acids, urea, and humic substances [3]. Urea, a smaller nitrogenous compound [7], has been reported to be higher in concentration in the coastal ecosystems [8]. It is has been observed that marine plants such as seagrass [9, 10] and algae (e.g. phytoplankton and macroalgae) [11, 12] use urea as a N source. However, for the absorption of organic N, both marine plants and algae need the assistance of microorganisms [13–15]. It has been found that microbiota associated with seagrass leaves can increase N uptake of seagrass by mineralizing amino acids [14]. Similarly, epiphytic bacteria of algae have found to promote algal growth by providing bioavailable N [16]. The source of bioavailable N mainly comes from the N_2_ fixation and the decomposition of organic matter, which are both carried out by specific microbial functional groups [17, 18].

The *ureC* gene is the largest of the genes encoding urease functional subunits and contains several highly conserved regions, which is suitable for use as PCR primer sites and widely used in urease analysis [19]. UPB, which contain the *ureC* gene, are a group of functional microorganisms and are widely found in various marine environment, such as in seawater [20, 21], marine sediments [22], and sponge surfaces [23]. They play a critical role in the N cycling of marine environments by converting organic N into inorganic N. Urease, produced by UPB, facilitates organic N decomposition and is found in various bacterial genera, including *Bacillus*, *Helicobacter*, *Proteus*, *Enterobacter*, *Pseudorhodobacter*, *Streptococcus*, *Escherichia*, *Lactobacillus*, *Enteroccocus*, *Weissella*, and *Mycobacterium*, etc. [21, 24–26]. Currently, there have been several studies on urease in marine environments. These studies include investigations on the diversity of UPB at the phylogenetic level, and on the role of urease in urea utilization at genetic level. For example, Siegl et al. [27] used single-cell genomics sequencing to identify a 10 ORF containing urease gene cluster from the *Poribacteria*, including various ABC-transporter, three urease subunits (*ureA*, *ureB*, *ureC*), and three accessory proteins. In another study, the transcriptional activity of the *ureC* gene and the phylogenetic diversity of bacteria with *ureC* gene were detected, providing insight into bacterial potential in urea utilization [23]. Despite the extensive studies on UPB in marine environments, investigations on UPB and the ecological functions of urease in the cultivation environment of macroalgae are lacking.

*G. lemaneiformis*, a member of the genus *Gracilariopsis*, is an economically important macroalgae and is widely distributes in coastal areas of China [6, 28]. It is the third-largest-mass-cultivated seaweed in China, following *Saccharina* and *Pyropia* [6], with a cultivation area of approximately 10,459 ha in 2020 (data from China Fishery Statistical Yearbook 2021). The large-scale commercial cultivation of *G. lemaneiformis* has gradually formed an influential seaweed field, which has produced a series of ecological effects [6]. For instance, it not only effectively improves the water quality of coastal environments but also contributes to increasing the marine carbon sink and mitigate climate change [29–31]. Although a significant number of studies have been conducted, mainly focusing on the diversity of epiphytic bacterial communities on *G. lemaneiformis*, and the microbial communities of seawater and sediment in the cultivation environment of *G. lemaneiformis* [18, 32]. However, the diversity of functional bacteria in the cultivation environment of *G. lemaneiformis* has not been thoroughly studied. Based on an in-depth analysis of 16S data, we discovered that the presence of a variety of UPB genera (such as *Lactobacillus*, *Escherichia-Shigella*, *Mesorhizobium*, *Helicobacter*, and *Streptococcus*) in the epiphytic bacterial communities of *G. lemaneiformis*. It is found that, due to the high productivity and the ability to absorb large quantity of N [28], *G. lemaneiformis* confronts the stress of inorganic N deficiency at the end of cultivation periods. In response, organic N (e.g. urea) can be used as a supplementary N source for the algae to utilize. Moreover, *G. lemaneiformis* is found to utilize urea as a N source to maintain its growth [7], but the role of functional bacteria found in the cultivation environment of *G. lemaneiformis* are poorly understood. Therefore, our goal was to explore the diversity and urease activity of UPB in the cultivation environment of *G. lemaneiformis*, their role in helping urea uptake by macroalgae, and their contribution to the marine N cycle. As to the best of our knowledge, these aspects have not been studied previously.

## Materials and methods

### Study area and sample information

The commercial seaweed cultivation area of Shen’ao Bay, Nan’ao Island involves large-scale cultivation of *G. lemaneiformis*, often accompanied by small-scale cultivation of *Porphyria haitanensis*. Samples of *G. lemaneiformis*, *P. haitanensis*, and seawater were collected from the algae cultivation field of Nan’ao Island (117°6′40″E, 23°29′9″N), Shantou, Guangdong Province, China in winter. The environmental conditions of the sample collection site were: water temperature 13.8 oC, pH = 7.8, salinity 30.5 ppt, dissolved oxygen 8.0 mg·L^−1^. The algal samples were stored in sterile polyethylene bags with surrounding seawater, while the seawater samples were stored in sterile polyethylene bottles. Both algal and seawater samples were stored at 4 oC and transported to the laboratory within 2 h for isolation of epiphytic and free-living bacteria.

### Isolation of epiphytic and free-living bacteria

In the laboratory, samples of *G. lemaneiformis* and *P. haitanensis* were washed three times with autoclaved seawater to remove loosely attached epiphytes, sand particles, and other attached settlements [33, 34]. After rinsing, firmly attached epiphytic bacteria from algae surface were swabbed with sterile cotton buds. The cotton bud heads containing epiphytic bacteria were cut using sterilized scissors and transferred to marine agar 2216 E plates using sterile tweezers. Subsequently, these cut portions were swabbed thoroughly onto the plates, with all operations conducted in close proximity to a flame. For free-living bacteria, a 0.1 mL seawater sample was dropped onto marine agar 2216 E plates and spread with spreader. The plates were then incubated at 28℃ for 3 days. Morphologically different bacterial colonies such as size, shape, color, were picked using an inoculating loop to make streak plates for getting purified colonies. This step was repeated twice in order to obtain pure individual colonies (19 colonies from *G. lemaneiformis*, 17 colonies from *P. haitanensis*, 5 colonies from seawater), which were then preserved at -80℃ in marine broth supplemented with 25% sterile glycerol.

### DNA extraction and 16S rDNA gene sequencing

The DNA of pure bacterial colonies was extracted by following the procedures described in the DNA extraction kit (TIANGEN Biotech, Beijing). The 16S rRNA gene was amplified using universal primers 27F (5’-AGAGTTTGATCMTGGCTCAG-3’) and 1492R (5’-TACGGYTACCTTGTTACGACTT-3’). PCR was carried out in a 30 μL reaction mixture containing 1 μL genomic DNA, 3 μL Buffer, 3 μL of each primer, 2 μL dNTP, 0.2 μL DNA Polymerase (Sigma), and 17.8 μL H_2_O with a thermal cycle of 95℃ for 5 min, 35 cycles of 30 s at 95℃, 30 s at 55℃, and 1 min at 72℃, followed by 72℃, for 10 min in a Bio-rad T100™ Thermal Cycler (Bio-Rad, USA). The quality of PCR products was verified by 1% agarose electrophoresis gel. Pure isolates were sequenced using the 3730XL DNA Analyzer (ABI, USA). The sequencing primers used for the 16S rRNA gene were V4-515F (5’-GTGCCAGCAGCCGCGGTAA-3’) and V4-806R (5’-GGACTACCAGGGTATCTAA-3’). The genetic relationship between different samples and known bacterial species were determined according to blast results. The most closely related bacterial species was selected as the species identification information of the sample. Different strains with identical description of matched species and accession number were considered to be the same species. The sequence data obtained from this study has been deposited in the NCBI GenBank database under the accession number SUB11205669. All data are available at https://submit.ncbi.nlm.nih.gov/subs/?search=SUB11205669.

### Identification of UPB and phylogenetic diversity analysis

The UPB were screened using a urea agar chromogenic media (10 mL), which contained 0.1 g tryptone, 0.1 g D-( +)-glucose, 0.2 g KH_2_PO_4_, 0.0012 g phenol red, 1.5 g agar, 2% urea solution (w/v), 0.1 L artificial seawater (ASW) [23]. The ASW used in this study consisted of 0.11 g CaCl_2_, 1.02 g MgCl_2_·6H_2_O, 3.16 g NaCl, 0.075 g KCl, 0.1 g Na_2_SO_4_, 0.24 g Tris–HCl, 0.002 g NaHCO_3_, 0.1 L dH_2_O (pH = 5.87). Fresh isolates were streaked on urea agar chromogenic media and cultured in an incubator at 28 ℃ for two days. The isolates were scored positive for urease activity if the color of media changed from pale yellow to pink/fuchsia/orange.

The *ureC* gene of UPB was amplified using universal primers L_2_F (ATHGGYAARGCNGGNAAYCC) and L_2_R (GTBSHNCCCCARTCYTCRTG) [23]. PCR was carried out in 20 μL with a thermal cycle of 98℃ for 30 s, 35 cycles of 10 s at 98℃, 30 s at 59℃ and 30 s at 72℃, followed by 72℃ for 2 min. PCR products were verified by 2% agarose electrophoresis gel and subjected to sequencing by using a 3730XL DNA Analyzer (ABI, USA). The primers used for sequencing of the *ureC* gene were the same as those used for amplification of the *ureC* gene.

The *ureC* gene sequence was translated to protein sequence (https://web.expasy.org/translate/). The *ureC* gene sequences of various UPB were blasted to check their sequence homology against other sequences from NCBI GenBank (https://blast.ncbi.nlm.nih.gov/Blast.cgi). The protein sequences of *ureC* gene of 16 known UPB with the highest homology were selected. The aligned protein sequences were used to construct the phylogenetic trees with the neighbor joining method using the MEGA 6.06 software. The sequences were compiled and aligned using ClustalW embedded in MEGA 6.06. For reliability, the bootstrap test was performed with 1000 replications in the phylogenetic trees [35].

### Urease activity of UPB

A 2 mL UPB bacterial culture was inoculated into 100 mL of fermentation media and cultured at 28℃ and 180 rpm for 24 h. The fermentation media contained 2% D-( +)-glucose, 1% tryptone, 0.5% yeast extract, 0.5% beef extract, 0.2% KH_2_PO_4_, 0.5% NaCl, 0.5% urea, 0.005% Ni(NO_3_)_2_, and 1000 mL ASW. The fermentation media was then centrifuged at 10,000 g for 20 min to separate the bacteria. The bacterial precipitates were suspended in 5 mL of PBS buffer and mechanically homogenized using ultrasonic cell pulverizer (30 W, 4 s/4 s) for 3 min on ice. Next, the supernatant was collected by centrifugation at 12,000 g for 5 min at 4℃. The protein concentration of the supernatant was determined using the Coomassie brilliant blue kit (A045-2–2, Nanjing Jiancheng Bioengineering Institute, Nanjing, China).

The urease activity was measured by using Berthelot reaction colorimetry method [36]. Briefly, a mixture of 2.5 mL 10% sterile urea solution, 5 mL PBS buffer (8.00 g NaCl, 0.20 g KCl, 1.44 g Na_2_HPO_4_, 0.24 g KH_2_PO_4_, pH = 7.4, constant volume to 1000 mL), and 10 μL crude enzyme solution were mixed thoroughly and incubated at 40℃ for 20 min. Then, 50 μL reaction solution, 400 μL sodium phenol solution, 300 μL 0.9% sodium hypochlorite solution were sequentially added to the reaction tube and incubated at 40℃ for 20 min. Finally, the OD_578_ value of the color-stabilized reaction solution was determined within 1 h. The control experiment was set up to replace 10 μL crude enzyme solution with an equal volume of heat-inactivated crude enzyme solution, other conditions remained unchanged. Incubation and determination were then carried out following the same procedure. The enzyme activity U was defined as that 1 U was equal to the release of 1 μmol NH_3_ after 1 mg enzyme solution reacted at 40℃ for 1 min.

### Co-culture of *G. lemaneiformis* and UPB

*G. lemaneiformis* was washed with sterile seawater and pre-cultured in modified f/2 medium for one week according to our previous study [37]. Given *G. lemaneiformis*' high productivity and its capability to absorb large quantities of nitrogen, it faces inorganic nitrogen deficiency stress in the middle and later stages of cultivation. To simulate this condition, we performed a 4-day incubation in f/2 medium with low N concentration (i.e. 5.49 μmol·L^−1^) after pre-culture. Prior to the experiment, strict bacterial removal of *G. lemaneiformis* was performed using previous reports with minor modifications [38, 39]. Briefly, *G. lemaneiformis* was immersed in 2 L modified f/2 medium for 4 h and sonicated in a SCIENTZ JY92-IIN Sonicator (Ningbo Scientz Biotechnology Co., Ltd. Ningbo, China) for 10 min (50W, 5 s/5 s). Then, *G. lemaneiformis* was incubated in 2 L modified f/2 medium containing a mixture of antibiotics, constituting 1.0 g of streptomycin sulfate, 1.0 g of penicillin G potassium, 1.0 g kanamycin sulfate, 1.2 mg nalidixic acid, 125 mg vancomycin hydrochloride. After a 24-h incubation, the *G. lemaneiformis* were washed three times with sterile seawater to remove residual antibiotics from the algal surface. Small portions (1–2 cm) of the algal bodies were cut using sterilized scissors and placed onto LB agar solid media, followed by incubation at 28℃ for 3 days. The colony formation on plates was observed to determine whether the algal body was sterile. Sterilized *G. lemaneiformis* showed minimal colony formation on the plates (Fig. S1), supporting the conclusion of basic sterility. The axenic *G. lemaneiformis* were divided into two groups (Table 1), each in triplicate. The UPB strain was inoculated in marine 2216 E liquid media and cultured overnight at 180 rpm under 28℃. The absorbance value can, indirectly reflect the concentration of bacterial solution, was utilized to guide the preparation of bacterial solution. The bacterial concentration required for this study was 2.5 × 10^8^ cells/mL, determined by measured value of bacterial OD_600_ [39]. Group-1 contained axenic *G. lemaneiformis* weighting 5.0 ± 0.05 g (fresh weight) and 1 mL UPB at the concentration of 2.5 × 10^8^ cells/mL. Group-2 contained simply 5.0 ± 0.05 g (fresh weight) axenic *G. lemaneiformis*. Group-3 contained simply 5.0 ± 0.05 g (fresh weight) *G. lemaneiformis*, which had not been sterile treated (defined as natural *G. lemaneiformis*). The CH_4_^15^N_2_O with the initial concentration of 75 μmol·L^−1^ was used as the sole N source for all groups in this study. All groups were maintained in 2 L modified f/2 medium and were incubated for 3 days in an illumination incubator (GXZ-500B, Ningbo Jiangnan Instrument Factory) at 20℃ with a light density of 100 μmol·m^−2^·s^−1^ and a light period of 14 h: 10 h (L/D).

**Table 1:** The design of co-culture experiment

Experimental type	Group	The status of *G. lemaneiformis*	UPB	Isotopic labeled material
Labeled Experimental Group	Group-1	axenic	*Oceanospirillum linum*	75 μmol·L^−1^ CH_4_^15^N_2_O
Labeled Control Group	Group-2	axenic	—	75 μmol·L^−1^ CH_4_^15^N_2_O
	Group-3	natural	—	75 μmol·L^−1^ CH_4_^15^N_2_O

*UPB* urease-producing bacteria. *Axenic* the surface of *G. lemaneiformis* was sterilized by antibiotics (streptomycin sulfate, penicillin G potassium, kanamycin sulfate, nalidixic acid, and vancomycin hydrochloride). *Natural* the surface of *G. lemaneiformis* was not sterilized by antibiotics. ‘—’ indicates that no UPB or other bacteria were used in these groups. The initial urea concentration of 75 μmol·L^−1^ refers to 0.9 mL 0.167 mol·L^−1^ of filtered sterilized urea solution was added into 2 L of f/2 medium.

### Measurement of urea content in medium

The urea content in the culture medium was measured following the method described by Revilla et al. [40]. Briefly, for the urea content measurements, 0.0288 mL of color development reagent and 0.144 mL mixed reagentIwere added to 1.0 mL water sample in a 1.5 mL centrifuge tube. The color development reagent consisted of 1:1 mixed color reagent (3.0 g diacetylmonoxime and 35 mg aminourea hydrochloride dissolved in 50 mL 50% [v/v] ethanol), and mixed reagentII (20 g MnCl_2_·4H_2_O and 0.4 g KNO_3_ were dissolved in 50 mL ddH_2_O). The mixed reagentIconsisted of 1:20 phosphate buffer (8 g NaH_2_PO_4_·2H_2_O was dissolved in 5 mL ddH_2_O), and 100 mL H_2_SO_4_. The tubes were incubated at 70℃ for 2 h. After incubation, the samples were cooled for 5 min on ice, and the absorbance at 520 nm was determined using a UV–visible spectrophotometer (UV2400, Sunny Hengping Instrument). The standard stock solution was prepared by dissolving 0.6 g of urea in 100 mL dd H_2_O (0.1 M urea), and the urea standard curve was drawn using a urea standard working solution (100 μM).

### The content of NH_4_^+^, urea, and total cellular N in *G. lemaneiformis*

The NH_4_^+^ content in *G. lemaneiformis* was spectrophotometrically analyzed as previously described by Meng et al. [41]. 100 mg powdered sample was extracted in 1 mL 100 mM HCl, and subsequently, 500 μL chloroform was added to each tube. The phases were separated by centrifugation (12,000 g, 10 min, 8℃) after rotating for 15 min at 4℃. The aqueous phase was transferred to a new 2 mL centrifuge tube containing 0.05 g activated charcoal, thoroughly mixed, and centrifuged at 20,000 g for 10 min at 8℃. The supernatant was transferred to a new 2 mL centrifuge tube and stored at -20℃. Next, 20 μL of the sample was mixed with 100 μL of phenol-sodium nitroprusside solution (1% [w/v] phenol and 0.005% [w/v] sodium nitroprusside were dissolved in water) and 100 μL of sodium hypochlorite-sodium hydroxide solution (1% [v/v] sodium hypochlorite and 0.5% [w/v] sodium hydroxide were dissolved in water). The mixture was incubated at 37℃ for 30 min, and light absorption was measured at 620 nm. The standard stock solution was prepared by dissolving 0.5349 g NH_4_Cl in 100 mL dd H_2_O (100 μM NH_4_Cl). The NH_4_^+^ standard curve was drawn by using NH_4_Cl standard working solution (1 μM).

The urea in *G. lemaneiformis* was extracted using the methods as described by Mérigout et al. [42]. Briefly, 1.5 mL of 10 mM ice-cold formic acid was added to 0.1 g of ground fresh sample. Each extract was vortexed for 15 min at 4℃ and centrifuged at 16,300 g for 15 min at 4℃, and the supernatant was transferred to a new 1.5 mL centrifuge tube. The determination of urea content in *G. lemaneiformis* followed the above methods in the measurement of urea content in the medium.

To determining the total cellular N in *G. lemaneiformis*, we followed the methods described by Barbarino and Lourenço [43]. Briefly, about 2 mg of dried powdered samples of macroalgae were weighed in small tin capsules and subjected to combustion at 1,150℃ for about 2 min in the combustion tube of a VarioEL/MICROcube elemental analyzer (Elementar Analysensysteme GmbH, Germany). The current pressure of oxygen pressure reducing valve and helium pressure reducing valve were 0.2, and 0.12 MPa, respectively. The reduction tube temperature was set at 850℃. The temperature of CO_2_ and H_2_O desorption column were set 20℃. The values were registered automatically by the recorder and integrator coupled to the analyzer.

### Isotope analysis of δ^15^N (‰, Atm-N_2_) in *G. lemaneiformis*

The frozen mixed ball mill (Retsch MM400, Verder Group) was used to grind the algal tissues of *G. lemaneiformis*, and the resulting powder samples were passed through a 100-mesh sieve. The samples and working standard samples were accurately weighed using an ultra-micro analytical balance (XP6, Mettler Toledo), and carefully packed into tin cup. Subsequently, these tin cups were placed into an automatic sampler plate in a specific sequence. The samples and working standard samples were burned at 1000℃ using an elemental analyzer (HT2000, ThermoFisher) to produce N_2_. The ^15^N and ^14^N ratios of N_2_ were detected by isotope ratio mass spectrometer (Delta V Advantage, ThermoFisher), and the δ^15^N values of the samples were calculated by comparing them with the international standard (Atm-N_2_). The total N content (N%) of the samples was calculated by comparing the peak area of samples with three working standard samples.

### Data statistics and analysis

The statistical analysis of physiological parameters among the groups was performed using repeated measures analysis of variance [5]. One-way analysis of variance was used to compare physiological parameters at the same point between different groups. All calculations and statistical analyses were carried out using SPSS 20.0 software, and the significant threshold was set to 0.05. All data were presented as means ± SD and were based on three biological replicates.

## Results

### Identification of bacterial isolates

A total of 41 single colonies were screened from *G. lemaneiformis*, *P. haitanensis*, and seawater, out of which 34 bacterial isolates were identified based on 16S rDNA gene. As shown in Table 2, at the phylum level, these isolates were classified into Proteobacteria (76.47%), Bacteroidetes (11.76%), Firmicutes (8.82%), and Actinobacteria (2.94%).

**Table 2:**
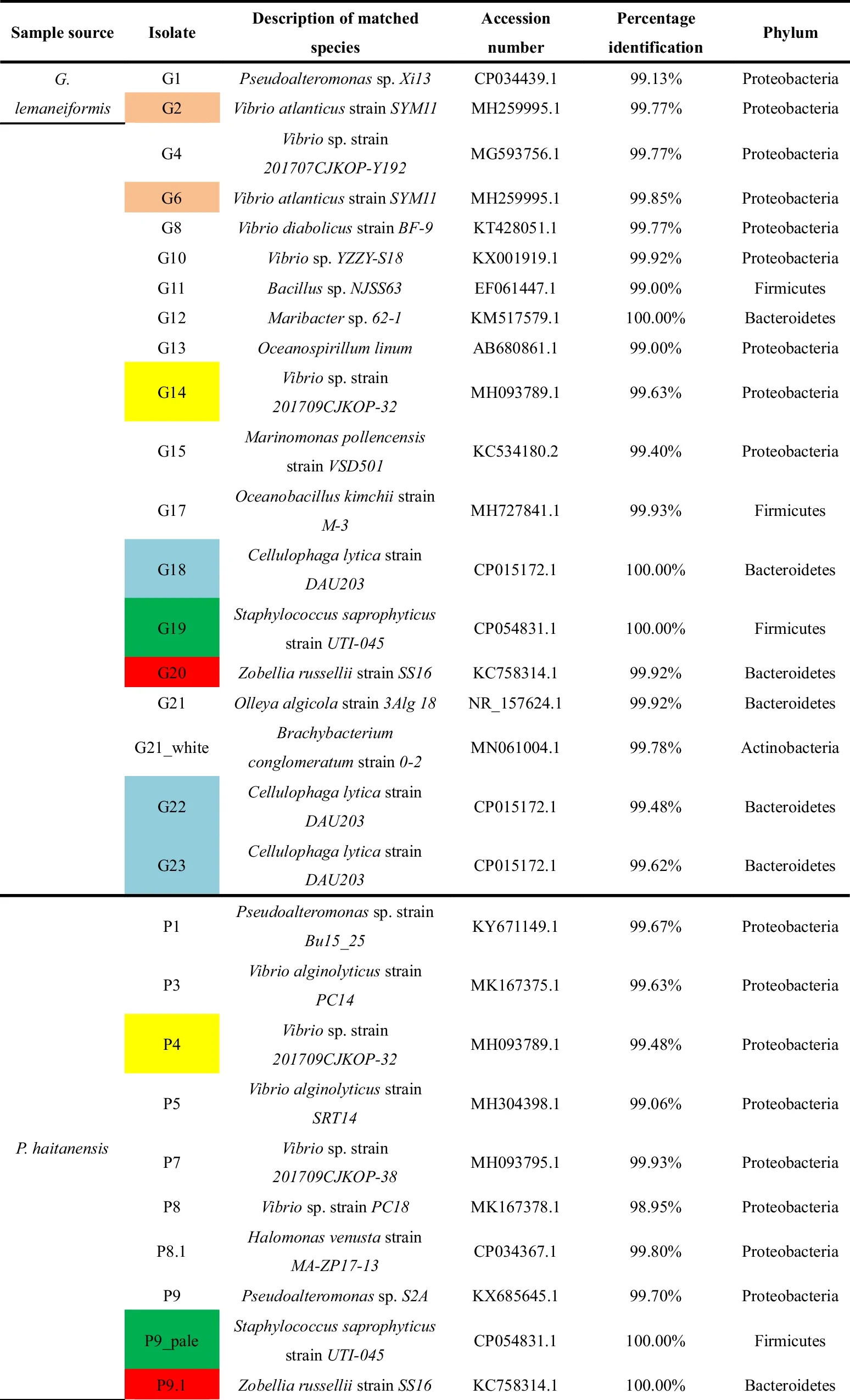

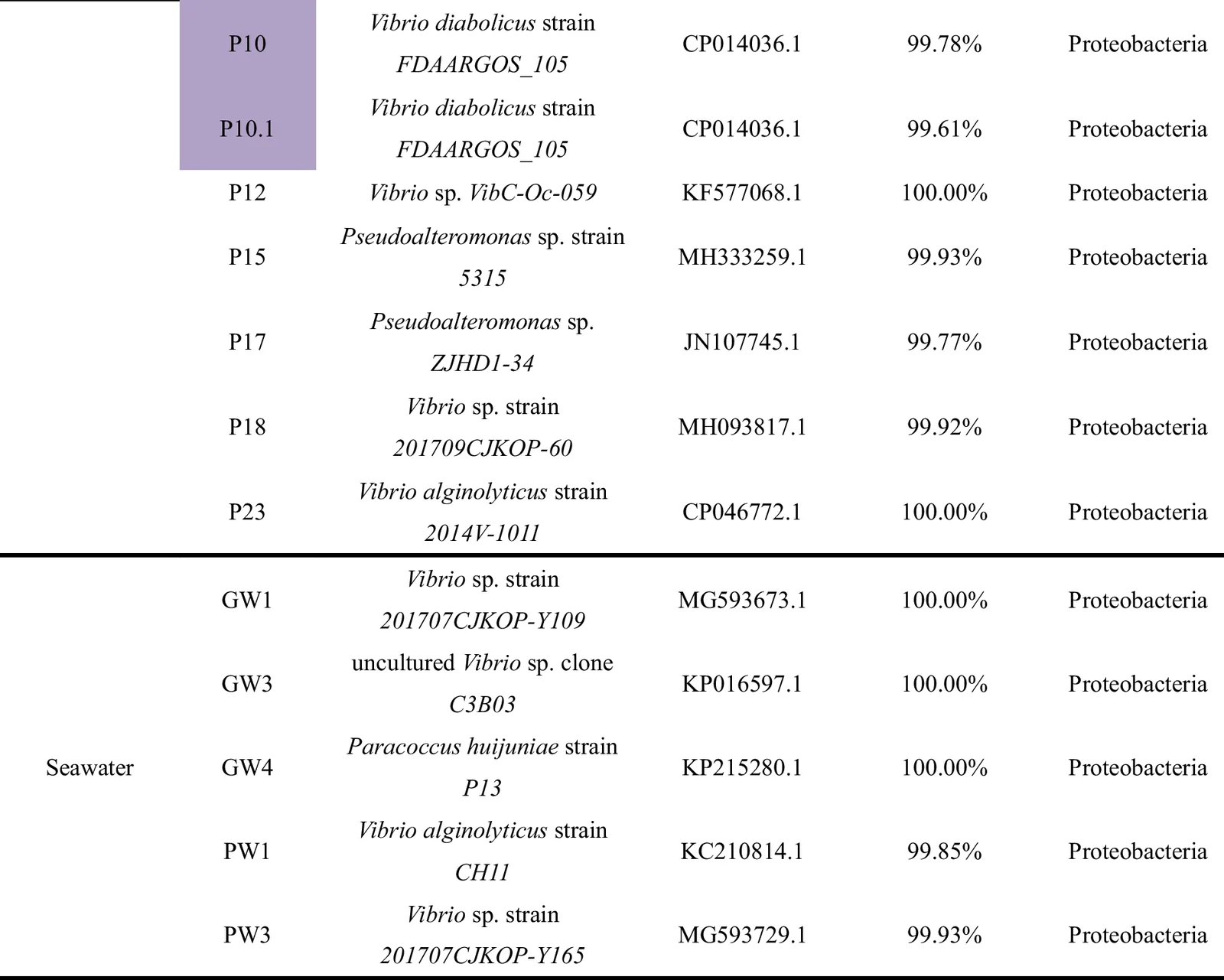
16S rRNA gene sequence identity of thirty-four bacterial species obtained from *G. lemaneiformis*, *P. haitanensis*, and seawater. The strains named after the “G + number”, “P + number”, “GW + number”, and “P + number” were isolated from *G. lemaneiformis*, *P. haitanensis*, seawater surrounding *G. lemaneiformis*, and seawater surrounding *P. haitanensis*, respectively.

Isolate labeled with same color belongs to the same species

### Screening of potential UPB

**Fig. 1:**
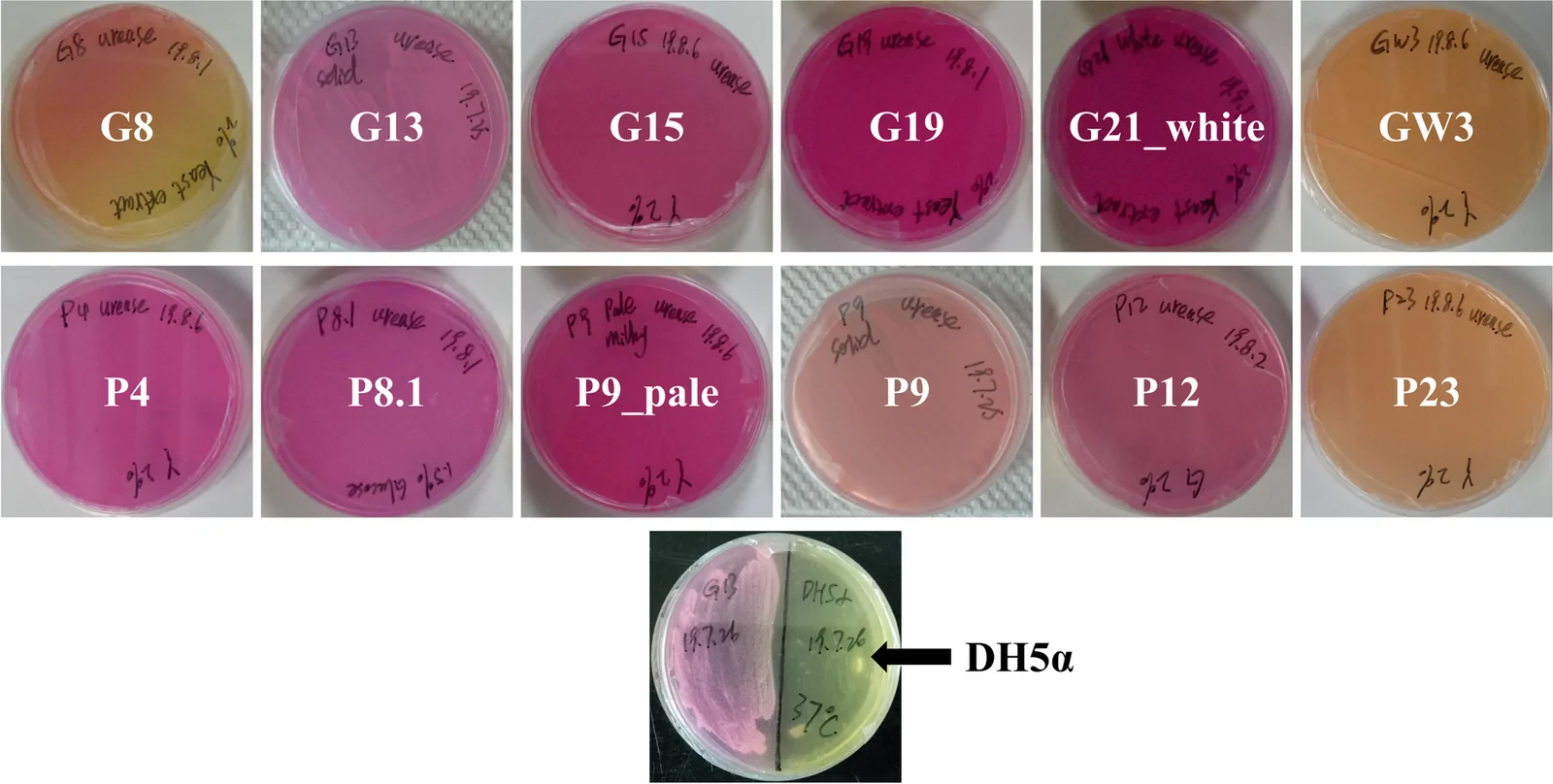
Chromogenic plate identification of UPB from the cultivation environment of *G. lemaneiformis*. Strains were scored positive for urease activity if the color of plates changed from pale yellow to pink/fuchsia/orange. DH5α: negative control for urease activity

A total of 12 bacteria were identified from the 34 bacterial isolates having the ability to break down urea (Fig. 1). The culturable bacteria in the cultivation environment of *G. lemaneiformis* were found to potentially contain urease, which we referred to as the potential UPB. In a previous study [20], the bacterium *Marinobacter litoralis*, which was isolated from the sponge, exhibited a similar color change in the urea agar chromogenic plate.

### Molecular identification and phylogenetical analysis of UPB

The target gene *ureC* was analyzed to identify urea-decomposing bacteria in various environments. The *ureC* gene target band of ten potential UPB (G21_white, G19, G15, GW3, G13, P4, P9_pale, P8.1, P12, and P23) except G8 & P9 was amplified by PCR and was about 400 bp in size (Fig. S2), consistent with the expected result. These strains with targeted bands were preliminarily believed to contain the *ureC* gene. To further verify the presence of *ureC* gene in these strains, 20 μL PCR product was sequenced using the L_2_F forward primer. Sequencing results showed that eight strains (G21_white, G19, G15, GW3, G13, P4, P8.1, and P12) were successfully sequenced, while two strains failed to be sequenced. Therefore, these eight potential strains were formally considered as UPB and classified into *Oceanospirillum* (G13), *Marinomonas* (G15), *Staphylococcus* (G19), *Brachybacterium* (G21_white), *Halomonas* (P8.1), and *Vibrio* (GW3, P4, P12) at the genus level. The urease gene (*ureC*) sequence details of all UPB are shown in Table S1. The amino acid sequence details of *ureC* gene of 8 UPB are shown in Table S1-1.

**Fig. 2:**
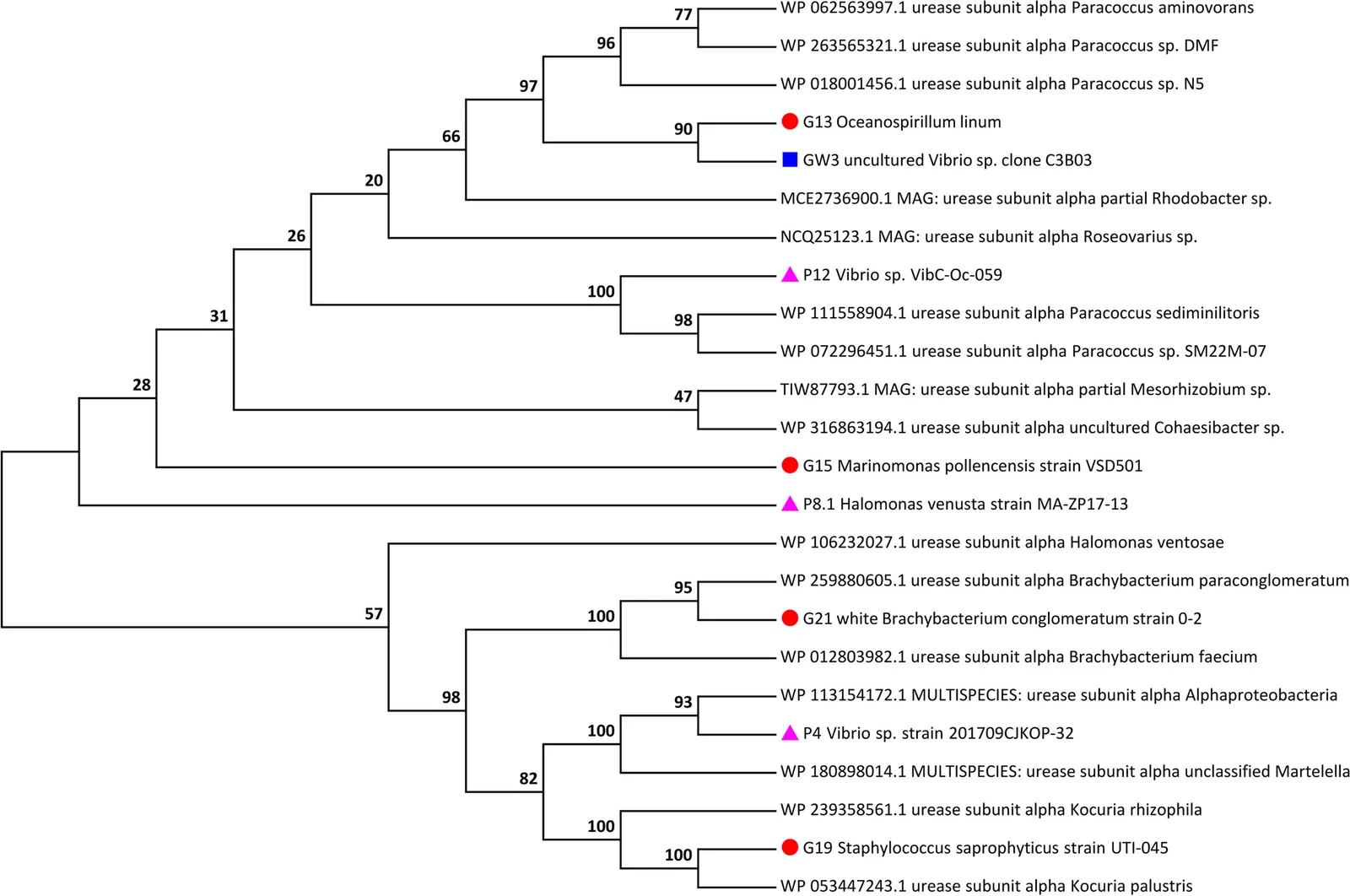
Phylogenetic tree of 8 various UPB (red dot, blue square, pink triangle) created with MEGA 6.06 using the Neighbor-Joining method

The phylogenetic tree constructed for the UPB strains represent their closest relatives as obtained from the Gene Bank (Fig. 2). The *ureC* gene sequences of G13 and GW3 were closely related to those of *Paracoccus* species, but were estranged from G19 and G21_white.

### Characteristic of urease activity from UPB

**Fig. 3:**
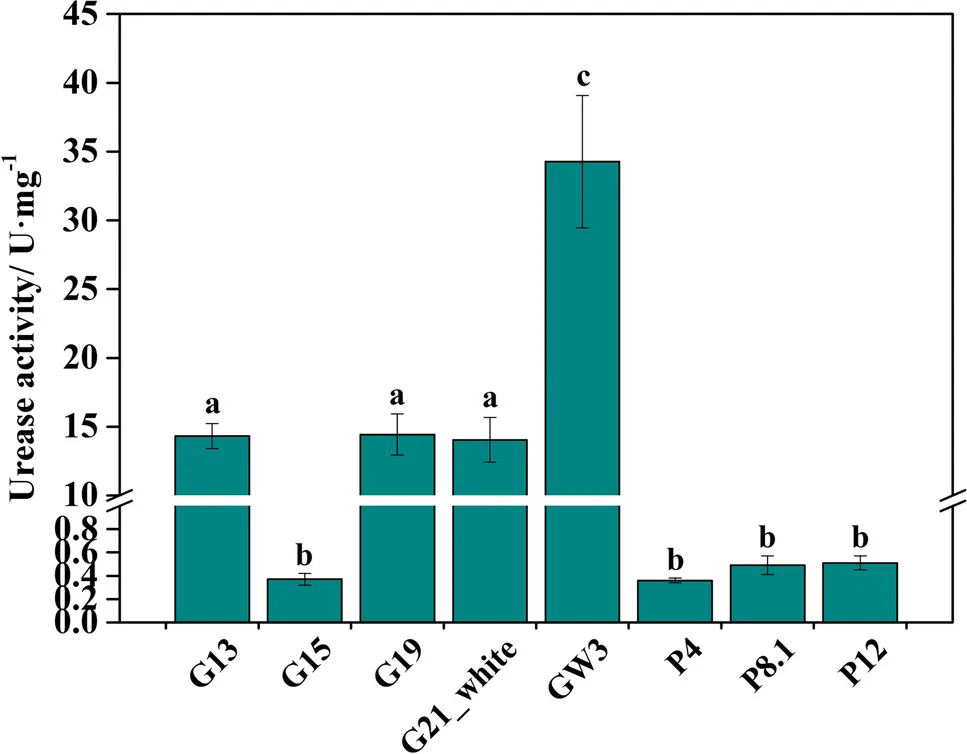
The urease activity of UPB from *G. lemaneiformis*, *P. haitanensis*, and seawater. Different letters (a, b, c) denote significant (*p* < 0.05) differences in urease activity between UPB. G13, G15, G19, and G21_white were isolated from *G. lemaneiformis*. GW3 was isolated from seawater surrounding *G. lemaneiformis*. P4, P8.1, and P12 were isolated from *P. haitanensis*

The urease activities of different UPB from *G. lemaneiformis*, *P. haitanensis*, and seawater are shown in Fig. 3. Among the epiphytic bacteria associated with *G. lemaneiformis*, the three UPB (G13, G19, and G21) exhibited urease activities higher than 10 U (1 U was equal to the release of 1 μmol NH_3_ after 1 mg enzyme solution reacted at 40 oC for 1 min) while G15 (*Marinomonas pollencensis*) showed lower activity. However, in the epiphytic bacteria associated with *P. haitanensis*, all UPB (P4, P8.1, and P12) demonstrated urease activities lower than 1 U. The strain with the highest urease activity (34.27 ± 4.81 U) was GW3, which was isolated from surrounding seawater of *G. lemaneiformis*.

### The urea consumption in medium

**Fig. 4:**
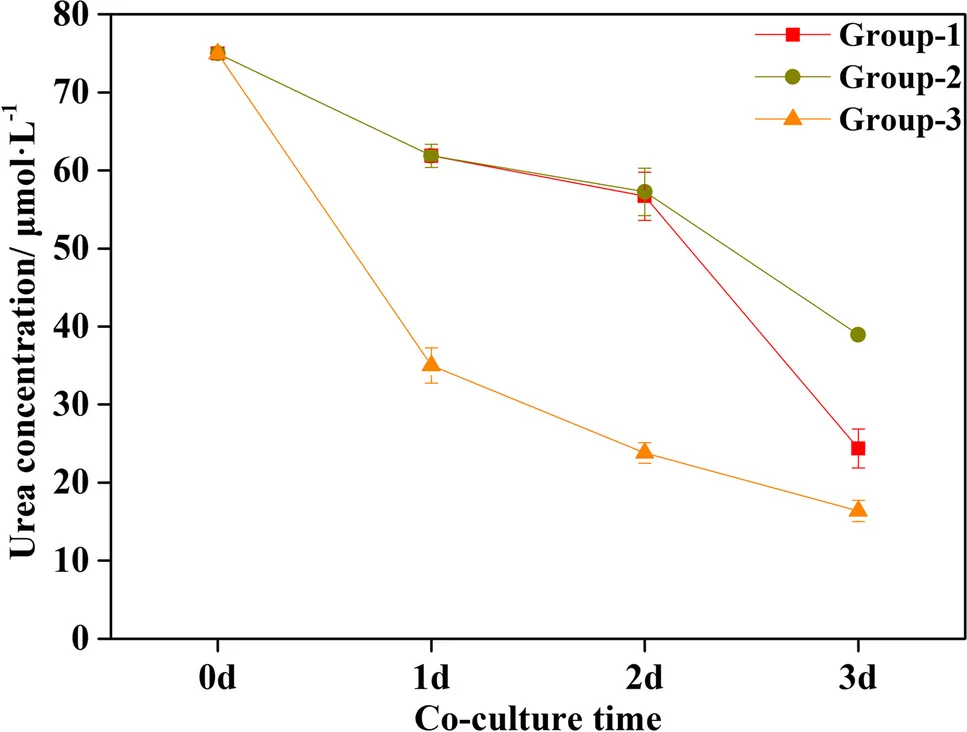
Dynamic urea consumption in medium. Group-1: axenic *G. lemaneiformis* with UPB in urea culture; Group-2: axenic *G. lemaneiformis* without UPB in urea culture; Group-3: natural *G. lemaneiformis* without UPB in urea culture. The initial urea concentration of Group-1, Group-2, and Group-3 was 75 μmol·L.^−1^

During the entire culture period, the urea absorption rates of Group-1 (sterilized macroalgae with UPB), Group-2 (sterilized macroalgae without UPB) had been on the rise, so the fitting of Michaelis–Menten equation did not converge (Fig. S3A & B). The maximum urea absorption rate of Group-3 (macroalgae with all epiphytes) was 0.191 ± 0.073 μmol·(g·d)^−1^ (Fig. S3C). There were no significant differences in urea content of medium between Group-1 and Group-2 on 1 d and 2 d (Fig. 4 & Table S2), indicating that the urea absorption rate in *G. lemaneiformis* was basically the same between Group-1 and Group-2 during the first 2 days. The urea content of medium in Group-1 on 3 d was 24.363 ± 2.515 μmol·L^−1^, which was significantly lower than that in Group-2 (38.956 ± 0.496 μmol·L^−1^) by 37.46% (Table S2, *p* < 0.001). While the urea absorption rate of Group-1 at 3 d was 0.385 ± 0.070 μmol·(g·d)^−1^, which was significantly higher than that in Group-2 by 120.40% (Table S3, *p* < 0.01). Moreover, the urea content of medium in Group-3 on 1 d was 35.021 ± 2.271 μmol·L^−1^, which was significantly lower than that in Group-1 and Group-2 by 43.41% (Table S2, *p* < 0.001). While the urea absorption rate of Group-3 at 1 d was 0.153 ± 0.013 μmol·(g·d)^−1^, which was significantly higher than that in Group-1 and Group-2 by 296.92% and 296.66%, respectively (Table S3, *p* < 0.001). Similarly, the urea content of medium in Group-3 on 2 d was 23.805 ± 1.311 μmol·L^−1^, which was significantly lower than that in Group-1 and Group-2 by 58.01% and 58.43%, respectively (Table S2, *p* < 0.001). While the urea absorption rate in Group-3 at 2 d was 0.107 ± 0.009 μmol·(g·d)^−1^, which was significantly higher than that in Group-1 and Group-2 by 335.30% and 392.21%, respectively (Table S3, *p* < 0.05).

### Physiological parameters of *G. lemaneiformis* cultured in different conditions

**Fig. 5:**
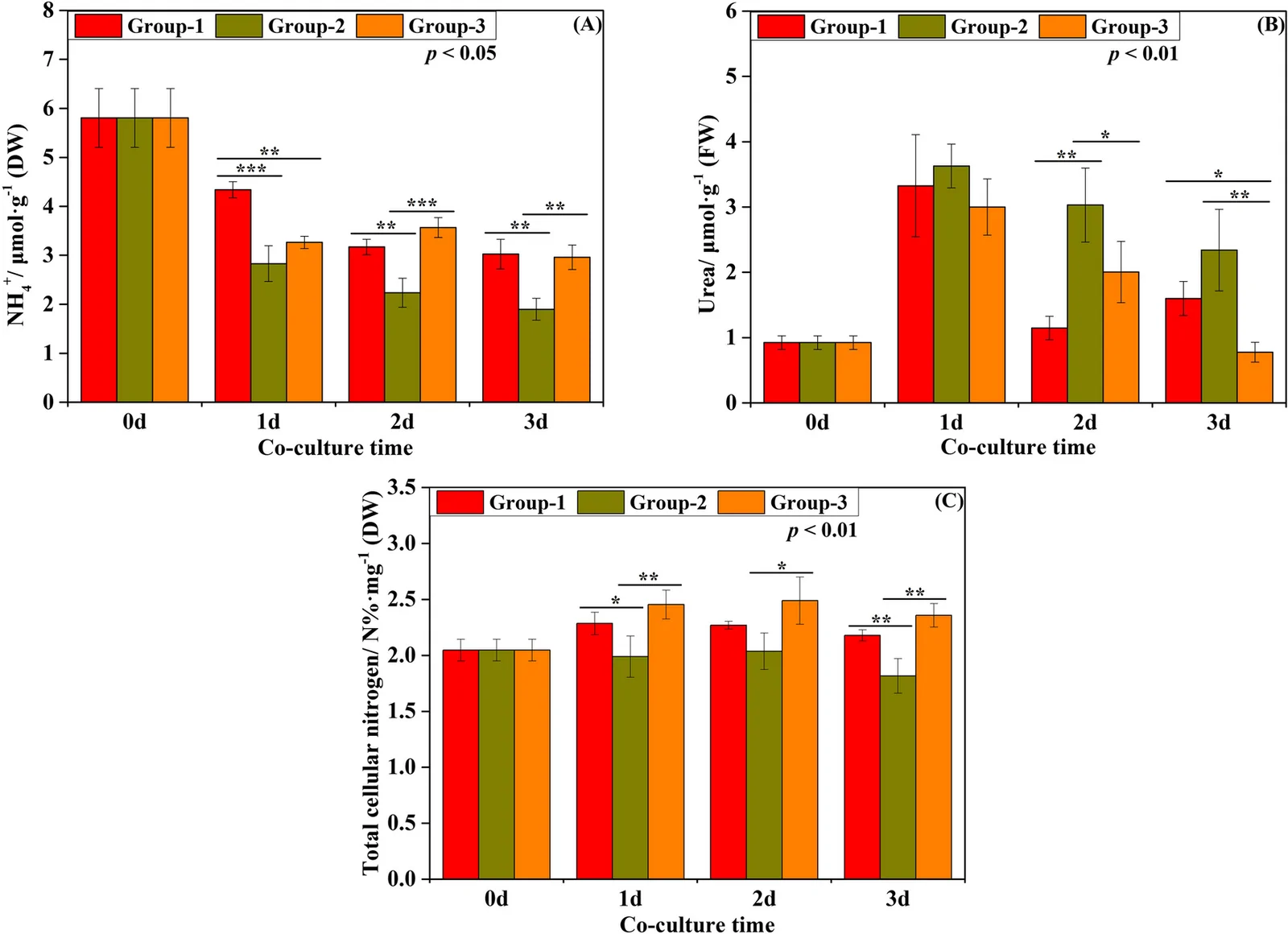
Physiological parameters of *G. lemaneiformis*. Group-1: axenic *G. lemaneiformis* with UPB in urea culture; Group-2: axenic *G. lemaneiformis* without UPB in urea culture; Group-3: natural *G. lemaneiformis* without UPB in urea culture. A: NH_4_^+^ content in *G. lemaneiformis*; B: urea content in *G. lemaneiformis*; C: total cellular N content in *G. lemaneiformis*. In all experiments, error bars indicate SD in three biological replicates and asterisks indicate significance between Group-1, Group-2, and Group-3. ‘*’ represents *p* < 0.05; ‘**’ represents *p* < 0.01; ‘***’ represents *p* < 0.001. The p-value corresponds to the statistical test of repeated measure ANOVA

To investigate the response of urea absorption by *G. lemaneiformis* to UPB, several key physiological parameters were measured throughout the experiment (Fig. 5). The NH_4_^+^ level of *G. lemaneiformis* in all three groups (Group-1, Group-2, and Group-3) decreased gradually with culture time. On day 1, 2, and 3, the NH_4_^+^ level of *G. lemaneiformis* in Group 1 were 4.340 ± 0.164, 3.170 ± 0.157, 3.024 ± 0.305 μmol·g^−1^, respectively, which were significantly higher than those in Group-2 by 34.82%, 29.43%, and 37.24%, respectively (Table S4, *p* < 0.001 for 1 d, *p* < 0.01 for 2 d and 3 d). The urea of *G. lemaneiformis* in Group-1, Group-2, and Group-3 increased to its highest point and then gradually decreased. There were significant differences in urea of *G. lemaneiformis* in Group-2 and Group-3 on 2 d and 3 d (Table S5, *p* < 0.05 for 2 d, *p* < 0.01 for 3 d). The total cellular N content of *G. lemaneiformis* in Group-1 was also measured, and we found 2.285 ± 0.100, 2.178 ± 0.050 N%·mg^−1^ on 1 d and 3 d, respectively, which were significantly higher than those in Group-2 by 13.00% and 16.57%, respectively (Table S6, *p* < 0.05 for 1 d, *p* < 0.01 for 3 d). In addition, the total cellular N of *G. lemaneiformis* in Group-3 was significantly higher than that in Group-2 during entire culture periods (Table S6, *p* < 0.01 for 1 d and 3 d, *p* < 0.05 for 2 d).

### Stable isotopic analysis of δ^15^N in *G. lemaneiformis*

**Fig. 6:**
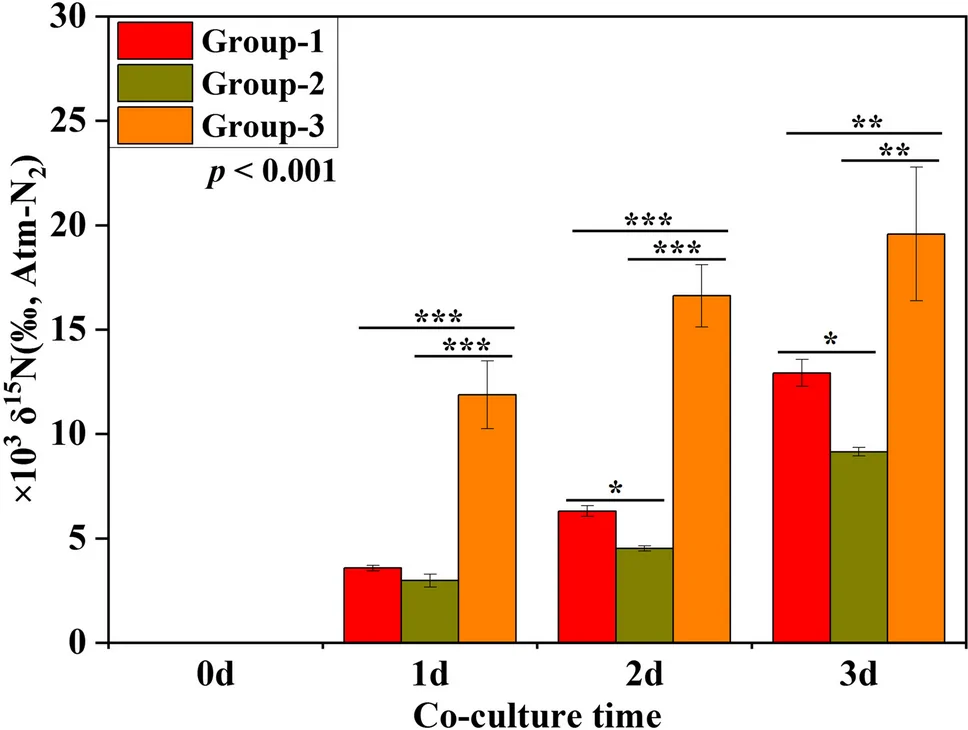
Mean (± standard deviation) δ^15^N isotopic values in *G. lemaneiformis* cultured in different conditions. Group-1: axenic *G. lemaneiformis* with UPB in urea culture; Group-2: axenic *G. lemaneiformis* without UPB in urea culture; Group-3: natural *G. lemaneiformis* without UPB in urea culture. In all experiments, error bars indicate SD in three biological replicates and asterisks indicate significance between Group-1, Group-2, and Group-3. ‘*’ represents *p* < 0.05; ‘**’ represents *p* < 0.01; ‘***’ represents *p* < 0.001. The p-value corresponds to the statistical test of repeated measure ANOVA

Our isotopic analysis results revealed that UPB or epiphytic bacteria associated with *G. lemaneiformis* facilitate algae uptake of δ^15^N derived from urea (Fig. 6). The variance analysis of groups showed that the δ^15^N accumulation in *G. lemaneiformis* were considerably different (F = 117.358, df = 2, *p* = 0.000, Repeated measures analysis of variance, Table S8) among the three groups. On 2 d and 3 d, the δ^15^N in *G. lemaneiformis* without microorganisms (Group-2) was 4513.842 ± 124.443 and 9142 ± 202.144 (‰, Atm-N_2_), which was significantly lower than those in *G. lemaneiformis* with UPB (Group-1) by 28.46% and 29.30%, respectively (Table S7, *p* < 0.05 for 2 d and 3 d). This indicates that the δ^15^N accumulation was greater in Group-1 compared to Group-2, with levels 1.398 and 1.414 times higher in Group-1 on 2 d and 3 d, respectively. Additionally, the δ^15^N in *G. lemaneiformis* with epiphytic bacteria (Group-3) on 1 d, 2 d, and 3 d were 11,878.620 ± 1622.051, 16,620.900 ± 1496.054, and 19,573.090 ± 3201.670 (‰, Atm-N_2_), respectively, which were significantly higher than those in Group-2 by 74.90%, 72.84%, and 53.29%, respectively (Table S7, *p* < 0.001 for 1 d and 2 d, *p* < 0.01 for 3 d). This demonstrated that the δ^15^N accumulation was greater in Group-3 compared to Group-2, with levels 3.984, 3.682, and 2.141 times higher in Group-3 on 1 d, 2 d, and 3 d, respectively.

## Discussion

### The diversity and ecology of culturable bacteria

The current study observed that a higher number of bacteria were isolated from the surface of macroalgae (*G. lemaneiformis* and *P. haitanensis*) compared to the surrounding seawater. Similar findings have been reported previously by Ismail et al. [44], where the number of strains isolated from seaweed capable of growth on marine agar was significantly higher than those isolated from the surrounding seawater. We assume that bacteria attached to the surface of algae are more likely to grow on Difco 2216 marine agar media than planktonic bacteria. One possible reason is that the harvested seaweed can be used as raw material for agar production [6], which makes it possible for these bacteria on algae are more likely to grow on agar media. Another possible reason is that adaptations evolved by bacteria on algae may enhance their ability to form colonies on artificial media [45]. Previous study points out that the survival strategies evolved by seawater bacteria, including responses to starvation, may dramatically reduce their ability to form colonies on nutrient-rich agar media [46]. Large portions of bacterial populations are dormant due to the reduction in the size and activity of bacteria led by starvation. In our study, most of the strains isolated from macroalgae and seawater belonged to Proteobacteria, and which is consistent with findings from Ali et al. [47], where the algae-associated and the planktonic communities were dominated by Proteobacteria. Specifically, the Gamma-proteobacteria were consistently recovered from the algae-associated community and were dominated by the *Vibrio*. This suggests that *Vibrio* belongs to the resident flora of *G. lemaneiformis* and *P. haitanensis*. As heterotrophs, *Vibrio* can readily use labile sugars derived from macroalgae as a carbon source to sustain their growth [48]. This could partly explain the high abundance of culturable *Vibrio* in the *Gracilariopsis* and *Porphyria* microbiome. In addition, the ecology of *Vibrio* has also been reported in other coastal ecosystems [49, 50]. For instance, in the ecosystem of coral reefs, Vibrionaceae with support of DOC released by algae, which ultimately dominate in bacterial communities [50]. The molecular structure of these compounds released by algae is one of the main factors affecting the microbial communities that metabolize them [49]. Interestingly, 35.29% of the strains isolated from the surface of *G. lemaneiformis* belonged to Bacteroidetes. Our findings are consistent with our previous study by Pei et al. (2021), where Proteobacteria (61.73%) was the most predominant phylum and Bacteroidetes (30.14%) was second dominant phylum on the surfaces of *G. lemaneiformis* in Nan’ao Island.

### The diversity of UPB and its functional studies

In the present study, 12 (41.38%) out of the total 29 isolates were determined to be positive for urease activity, and ten potential UPB except G8 & P9 were successfully amplified with *ureC* gene target band. This indicated that G8 and P9 did not contain *ureC* gene, but they were able to hydrolyze urea normally, suggesting that the presence of other bio-enzyme, e.g. urea amidolyase (UA), which is comprised of urea carboxylase (UC) and allophanate hydrolase (AH). These are widely distributed in fungi, bacteria, and other microorganisms, and play important roles in N cycling of the biosphere [25, 51, 52]. Eight potential UPB were formally identified as UPB by detecting the *ureC* gene. To the best of our knowledge, this is the first study to isolate culturable bacteria with urease activity from cultivation environment of *G. lemaneiformis* using the urea agar chromogenic medium. Moreover, the higher proportion (more than 25%) of UPB suggests that there were a large number of UPB in the cultivation environment of *G. lemaneiformis*. Senthil et al. [53] showed that the UPB isolated from samples of water and sediment in coastal area are identified as *Klebseilla* spp, *Proteus* spp, *Lactobacillus* spp, *Streptococcus* spp, and can be used for industrial production of urease. In our current study, UPB isolated from the cultivation environment of *G. lemaneiformis* belong to *Oceanospirillum*, *Marinomonas*, *Staphylococcus*, *Brachybacterium*, *Halomonas*, and *Vibrio*, which differ from previous study. This can be explained by the environmental specificity and abundant diversity of UPB. In the marine environment, the key steps of the N cycling process are mainly driven by microorganisms containing specific functional genes [54]. *G. lemaneiformis* is an commercially important macroalgae and widely distributes in coastal areas of China [6]. Therefore, we assume that diverse UPB in the cultivation environment of *G. lemaneiformis* play an important role in the N cycling of the coastal marine ecosystem.

### The structure and activities of UPB

Our study showed that there was a significant difference in urease activity among the various bacterial strains (Fig. 3), which could be attributed to the variations in bacterial urease structure. Bacterial urease proteins are comprise of both structural proteins and accessory proteins [55]. The structural proteins are mainly encoded by *ureA*, *ureB* and *ureC* genes. However, the trimer structure formed by the γ, β, and α subunits encoded by the *ureA*, *ureB*, and *ureC* gene represents Apo-urease, which lacks urease activity [56]. Thus, the activation of most bacterial urease typically requires the involvement of several accessory proteins [55], which are encoded by *ureD/H*, *ureF*, *ureG*, and *ureE* genes [56]. In the present study, some UPB (G15, P4, P8.1, and P12) isolated from *G. lemaneiformis* and *P. haitanensis*, showed low or undetectable urease activity, which probably due to the lack of some accessory proteins to activate urease. Conversely, the urease activities of other four UPB strains (G13, G19, G21_white, GW3) varied, possibly due to variations in the *ureC* gene sequence (Fig. 2). Previous studies have shown that variations in the *ureC* gene sequence in bacteria can lead to differences in bacterial urease structure [57, 58]. In addition to bacterial urease structure, various environmental factors can also affect the urease activity. For instance, the urease activity is positively correlated with urea concentration but negatively correlated with inorganic N concentration [59, 60]. This means that urease functions depending on the concentration of different forms of N in the environment. A previous study has also shown that the availability of N can influence the urease activity of bacteria by modulating *ureC* gene transcription [59]. Thus, various environmental factors could guide the function of bacterial urease or regulate the expression of urease gene, ultimately affecting the urease activity. In conclusion, the bacterial urease activity is not solely dependent on its structure but is also influenced by environmental factors.

### Effects of UPB on urea utilization in *G. lemaneiformis*

The experiment of co-culture of UPB and axenic *G. lemaneiformis* was designed to explore the response of urea uptake by G. *lemaneiformi*s. Our data showed that the urea consumption by *G. lemaneiformis*, which was aseptically treated (i.e. Group-2), decreased, but significantly increased after the addition of UPB (i.e. Group-1) (Fig. 4). Moreover, the urea consumption by *G. lemaneiformis* without aseptic treatment (i.e. Group-3) was higher than that by Group-1 and Group-2 (Fig. 4). After adding *Oceanospirillum linum* bacterium in Group-1, a part of urea was decomposed by urease secreted possibly by UPB, and thus the urea consumption of Group-1 was obviously higher than that of Group-2. Most organisms, and even microbial symbionts, that use urea as a N source rely on urease [23]. Ammonium was not only utilized by UPB, but also by *G. lemaneiformis*, resulting in the NH_4_^+^-N content and the percentages of total cellular N of *G. lemaneiformis* in Group-1 being higher than that in Group-2 (Fig. 5A & C). The increase of urea in *G. lemaneiformis* at 1 d (Fig. 5B) may be due to direct uptake of the urea molecule by algae itself and temporary storage in the algae [12]. After 2 d, urea in *G. lemaneiformis* began to decrease (Fig. 5B), indicating that urea would be transformed into a useable form after entering the algae to maintain the normal life activity of the algae [12]. It is worth noting that *G. lemaneiformis* still normally used urea without the participation of microorganisms (Figs. 4 & 6). Our findings are consistent with the previous study conducted by Tarutani et al. [61], which showed direct utilization of DON by *Ulva pertusa* without associated microorganisms. Moreover, Engeland et al. [9] also proposed that even after removal of epiphytes, phytoplankton, and partial bacterial communities, macrophytes could immediately absorb N from organic sources. These studies provide a scientific basis for differentiating between direct DON uptake and uptake after remineralization by the bacterial community. In addition, DON can be used by many phytoplankton directly through processing mechanisms, such as urease activity and amino acid oxidation [62, 63]. Our previous study (data not published) showed that UA sequences were found in the transcriptome data of *G. lemaneiformis*, indicating *G. lemaneiformis* has the potential ability to utilize organic N. The importance of organic N to macroalgal N depends on the availability of both dissolved inorganic and organic N compounds [64]. In this study, *G. lemaneiformis* was cultured in an organic N source (urea) after 4 days of low inorganic N culture, and urea would be the only N source available for *G. lemaneiformis* to absorb and utilize. Tyler et al. [65] have demonstrated that there were a relatively small fraction of amino acids and urea was assimilated by macroalgae when the inorganic N supply in environment was high. However, when dissolved inorganic N was low, organic compounds played a much more important role [65].

### The ^15^N accumulation in algae by isotopic analysis

In addition, the ecological function of urease has not been fully evaluated at the physiological level, so it is necessary to use stable isotopic tracer technique to track the ^15^N accumulation in macroalgae. To the best of our knowledge, this is the first study that used stable isotopic tracer technique to evaluate the effects of UPB on urea utilization in *G. lemaneiformis*. Evidence that UPB associated with *G. lemaneiformis* facilitate algae uptake of ^15^N derived from urea was provided by our results (Fig. 6). During entire culture time, there was greater ^15^N accumulation in *G. lemaneiformis* with microorganisms (regardless of whether UPB or epiphytic bacteria) compared to those where microorganisms had been removed. These results clearly indicated that macroalgae can absorb more organic N sources in the presence of UPB or epiphytic bacterial flora especially under inorganic N stress which is proved previously [61]. It was worth mentioning that Group-3 had a significantly higher uptake of ^15^N than Group-1 (Fig. 6), suggesting that a population of epiphytic bacteria that originally existed on the surface of algae could more improve urea uptake efficiency of *G. lemaneiformis* rather than adding a single UPB strain. Tarquinio et al. [14] reported that ^15^N accumulation was greater in leaves of seagrass with an associated microbiota compared to those where microorganisms had been removed, with levels 4.5 times higher in leaves with microorganisms by 12 h. In fact, mineralization of organic matters by seagrass associated microorganisms may increase the availability of N for uptake by seagrass [13, 15]. Bulk isotope analysis may not support specific isotope tracer accumulation point [14]. Therefore, high-resolution secondary ion mass spectrometry (NanoSIMS) will be considered to trace the uptake of ^15^N derived from urea for future studies. The direct or indirect uptake of organic N in many phytoplankton and microbes has been well documented [8, 66] but not for macroalgae. Smith et al. [12] reported that rates of urea uptake calculated by ^15^N enrichment were approximately two fold higher than those based on ^13^C enrichment, demonstrating direct uptake of the urea molecule in giant kelp. Therefore, the double isotope labeling method is of great significance for exploring the contribution ratio of microorganisms to the uptake of organic N by algal host. The direct uptake of urea molecule by macroalgae is likely benefit by a urea-transporting protein called DUR3-like [67, 68]. DUR3 proteins mediate high-affinity transport of exogenous and endogenous urea [68]. Based on the above reports, we assume that the macroalgae *G. lemaneiformis* can directly absorb urea via DUR3-like transporter. Decomposition of organic N by associated microorganisms, especially UPB, may enhance the availability of N for uptake by *G. lemaneiformis*.

## Conclusion

Eight UPB strains carrying the *ureC* gene were isolated, screened, and identified from the cultivation environment of *G. lemaneiformis*. The urease activity of these UPB varied, indicating differences in their ability to degrade urea. In the algae-bacteria co-culture, the physiological analysis showed significant increase in urea consumption in the culture medium having UPB or epiphytic bacteria compared to the axenic culture. Although *G. lemaneiformis* could utilize urea without the participation of microorganisms, the presence of UPB or epiphytic bacteria promoted the urea uptake. Our results also showed a significant increase in the total N content in the tissues of *G. lemaneiformis* with the existence of UPB or epiphytic bacteria compared to axenic culture. Stable isotope labeling was employed to track the accumulation of N in algae. It was found that the δ^15^N isotope values in algae increased markedly with the presence of UPB or epiphytic bacteria, further revealing that urea is utilized by *G. lemaneiformis*, and UPB or epiphytic bacteria promoted its utilization. In conclusion, the results of this study demonstrate that *G. lemaneiformis* uses organic N, and the presence of functional microorganisms on its surface significantly enhances to its N absorption capacity. These finding have not only important implications for understanding the ecological interactions between macroalgae and associated microorganisms, but also have significant implications for the marine N cycle.

## Supplementary Information

Below is the link to the electronic supplementary material.

Supplementary file1 (DOC 3689 KB)

## Data Availability

The datasets presented in this study are readily available in online repositories. For detail on the names of the repository/repositories and accession number(s) please refer to the article/Supplementary Material.
