# Supplementary material for: Diversity and ecological function of urease-producing bacteria in the cultivation environment of Gracilariopsis lemaneiformis
Source: Microb Ecol. 2024 Jan 23;87(1):35. doi: 10.1007/s00248-023-02339-y (PMC10806000; doi:10.1007/s00248-023-02339-y)
Supplement: Supplementary file 1 — Supplementary file1 (DOC 3689 KB) [file 248_2023_2339_MOESM1_ESM.doc]

**Diversity and ecological function of urease-producing bacteria in the cultivation environment of *Gracilariopsis lemaneiformis***

**Pengbing Pei^a,b^, Muhammad Aslam^a,d^, Hui Wang^a^, Peilin Ye^a^, Tangcheng Li^c^, Honghao Liang^a^, Qi Lin^b^, Weizhou Chen^a^, Hong Du^a,*^**

**^a^** Guangdong Provincial Key Laboratory of Marine Biotechnology, College of Science, Shantou University, Shantou 515063, China.

**^b^** Key Laboratory of Cultivation and High-value Utilization of Marine Organisms in Fujian Province, Fisheries Research Institute of Fujian, Xiamen 361000, China.

**^c^** Guangdong Provincial Key Laboratory of Marine Disaster Prediction and Prevention, College of Science, Shantou University, Shantou 515063, China.

**^d^** Faculty of Marine Sciences, LUAWMS, Lasbela 90150, Pakistan.

*** Corresponding author:** Hong Du, [hdu@stu.edu.cn](mailto:hdu@stu.edu.cn)

***Supplementary Information***

**2.6. Co-culture of *G. lemaneiformis* and UPB**


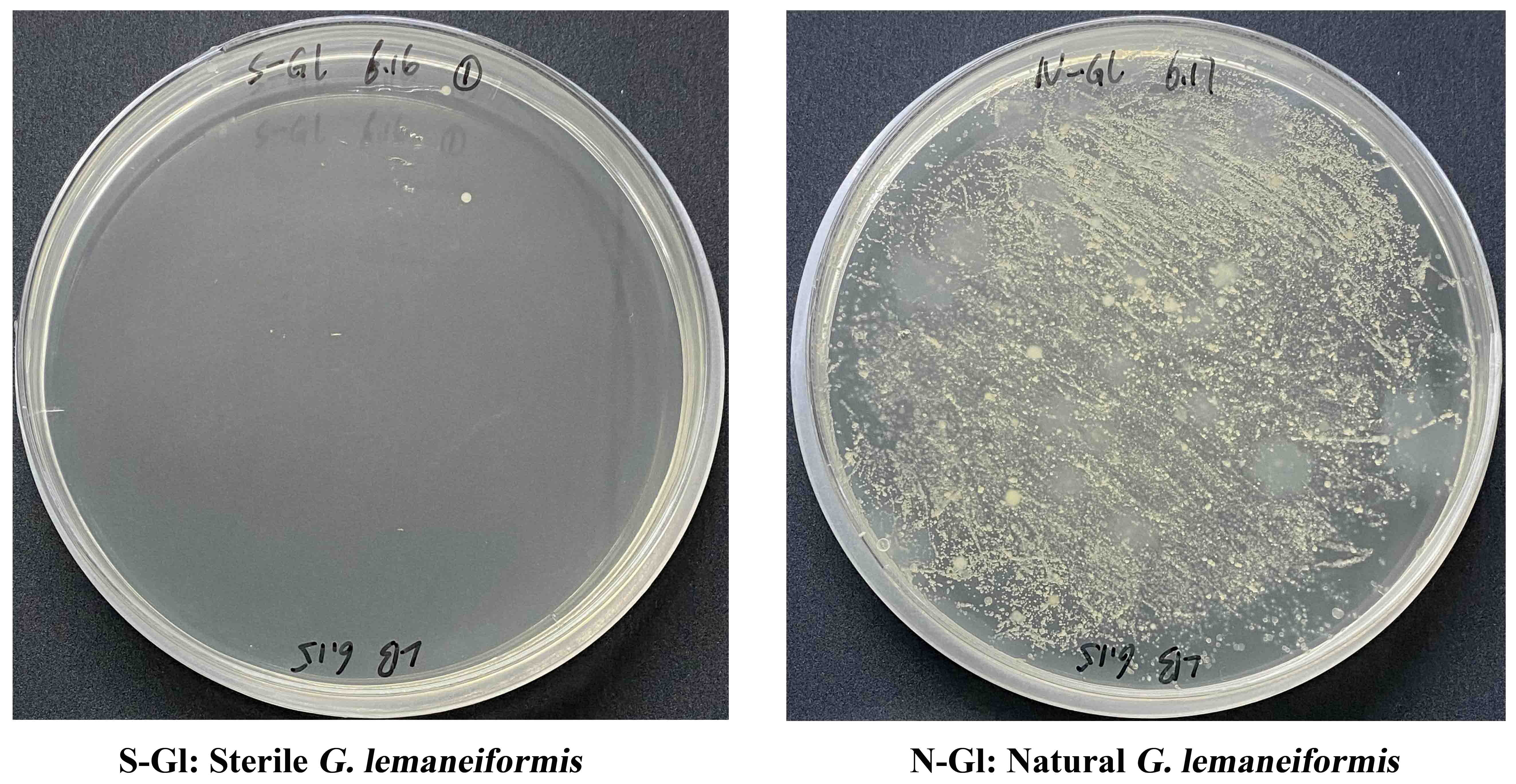


**Supplementary Figure 1.** Examination of sterile status of *G. lemaneiformis* by isolation method.

**3.3. Molecular identification of UPB**


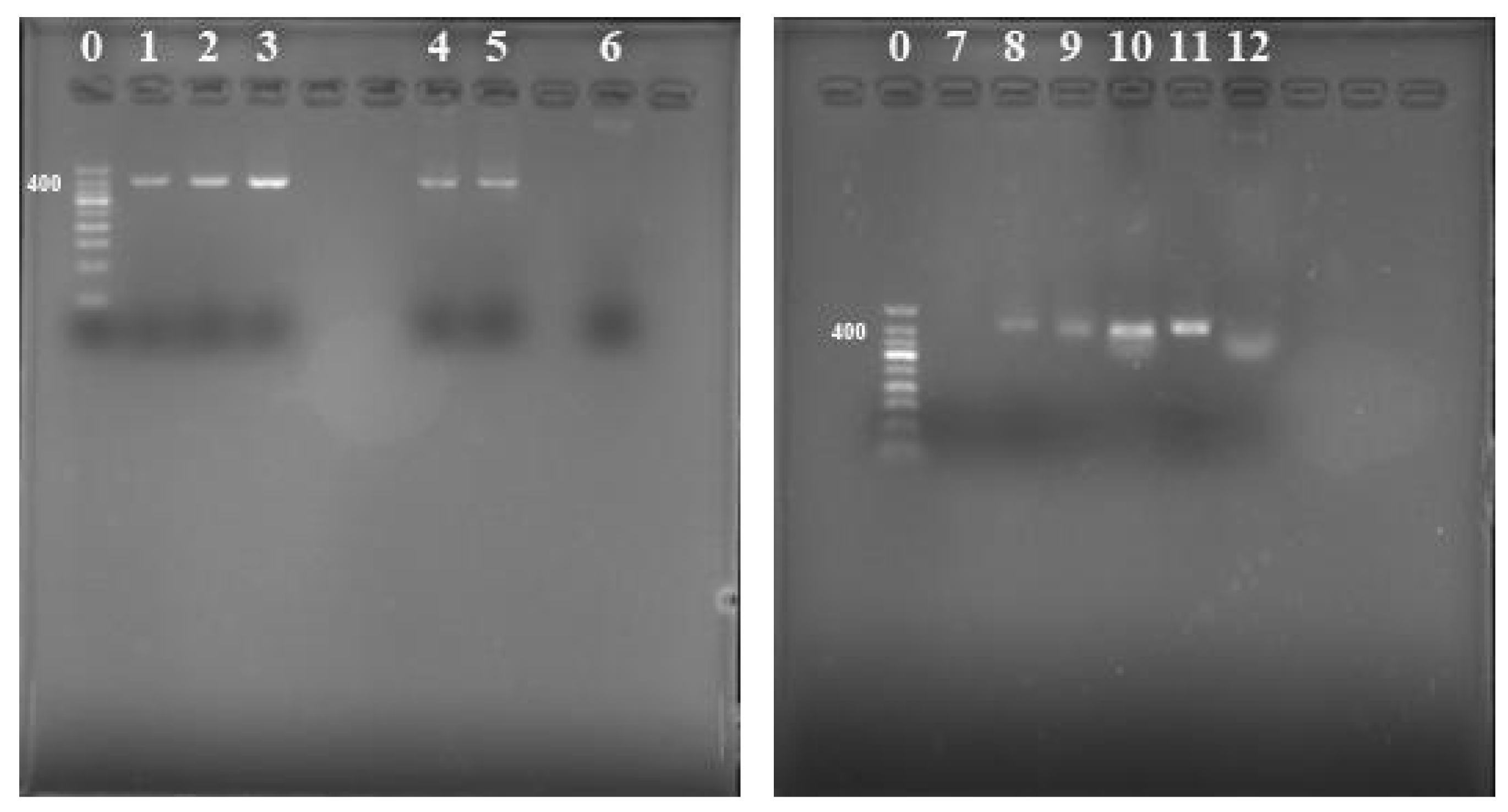


**Supplementary Figure 2.** The agarose gel electrophoresis of amplified *ureC* products by direct PCR. 0: 50 bp DNA Ladder; 1-12 are strain G21_white, G19, G15, GW3, G13, P9, G8, P4, P9_pale, P8.1, P12, and P23, respectively.

**3.5 The urea consumption in medium**


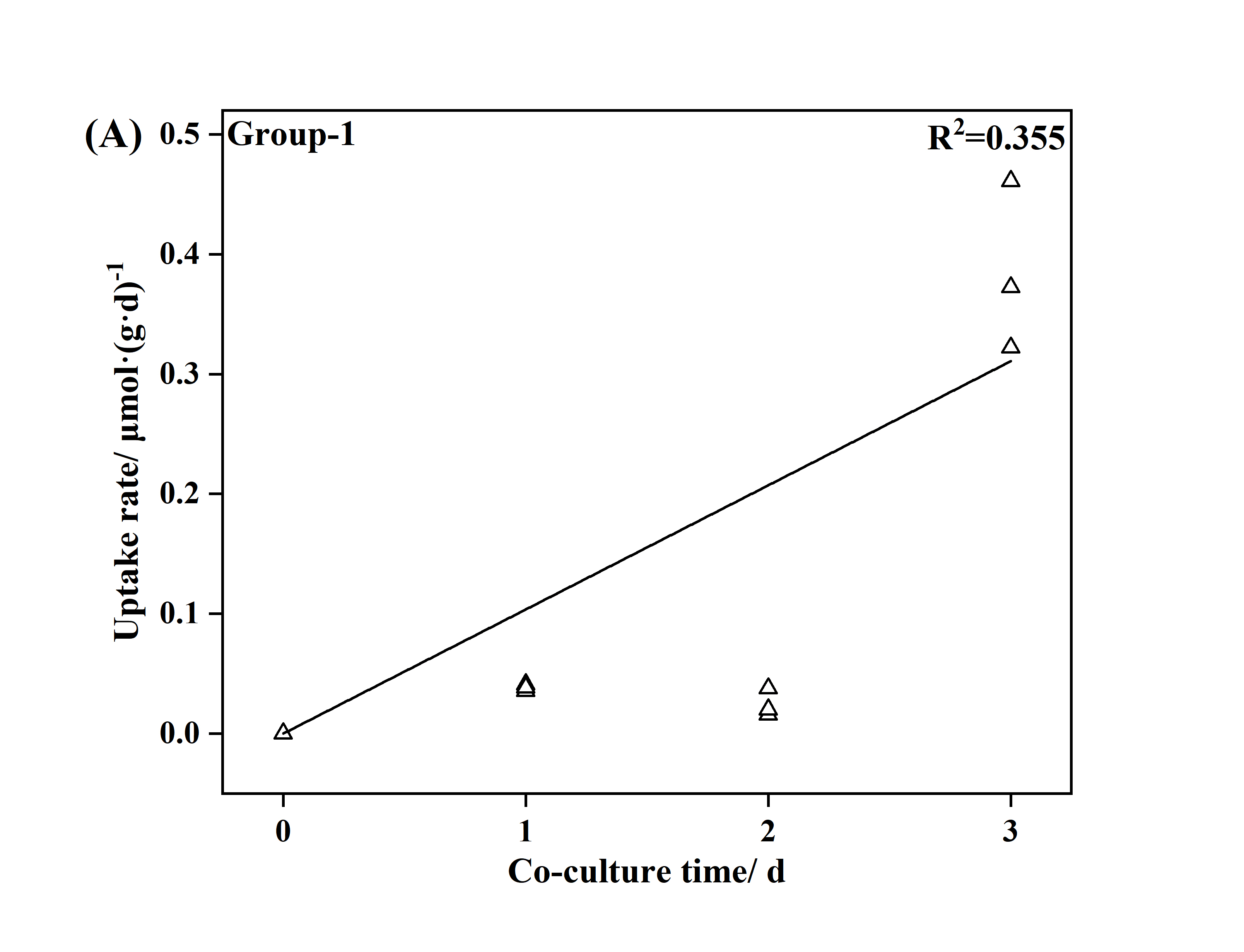

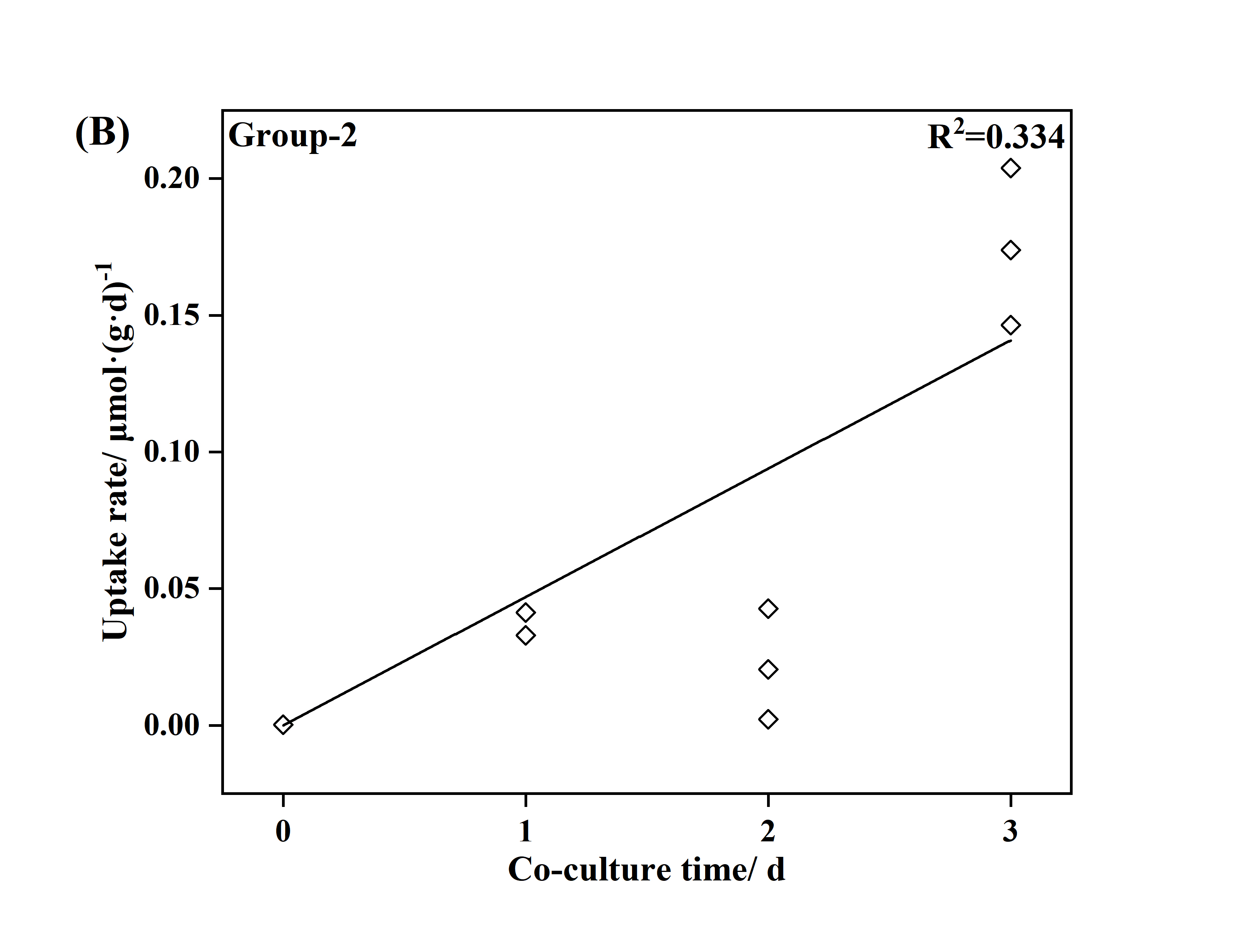


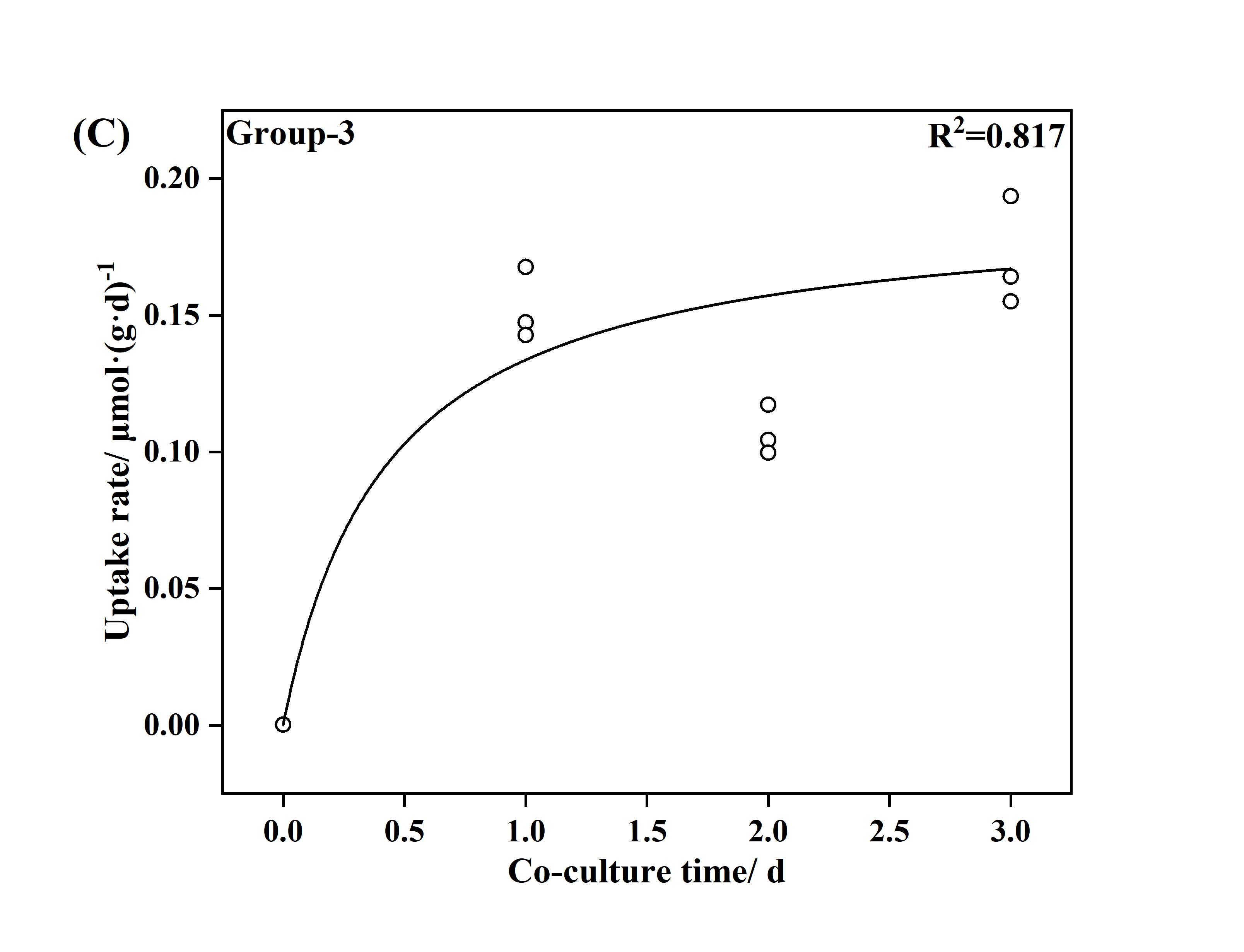


**Supplementary Figure 3.** The urea uptake rate in different groups. A: Group-1 (sterilized macroalgae with UPB); B: Group-2 (sterilized macroalgae without UPB); C: Group-3 (macroalgae with all epiphytes).

**Supplementary Table 1.** The urease gene (*ureC*) sequence of eight different UPB. G13: *Oceanospirillum linum*; G15: *Marinomonas pollencensis*; G19: *Staphylococcus saprophyticus*; G21_white: *Brachybacterium conglomeratum*; GW3: uncultured *Vibrio* sp.; P4: *Vibrio* sp.; P8.1: *Halomonas venusta*; P12: *Vibrio* sp..

| **Isolate** | **Urease gene (*ureC*) sequence** |
| --- | --- |
| G13 | GGCGACGGCGTGAGTGCTGATCGGCCCGGCACCGAGATCATCGCGGGCGAGGGCCGCATCCTGACCGCGGGCGCCATGGACGCGCATATCCATTTCATCTGCCCGCAGCAGATCGACGATGCGCTGCATTCGGGCATCACCACCATGCTGGGCGGCGGCACCGGCCCGGCGCATGGCACGCTGGCCACGACCTGCACCCCCGGCCCTTGGCATATCGGCCGCATGATGCAGGCCGCCGATGCCTTGCCGATGAACCTGGCCTTTGCCGGCAAGGGCAATGCCGCGCTGCCCGCCGCGCTGGAGGAACAGGTCCGCGCCGGGGCCTGCGCGCTGAAGCTGCACGAGGACTGGGGGCACAACA |
| G15 | GGCATCATGTCGATTCTCCTTTGGACAGGTACCGAGGTCATTGCAGGGGAGGATCCATCCTCACCGCTGGCGGCTTTGACGCTCACATTCACTTTATTTGCCCACAGCAGATTGAAGAAGCATTGCATTCTGGTGTGACCACCTGGATTGGCGGTGGCACCGGGCCTGCGCATGGCACCCTTGCCACCACTTGTACCCCCGGACCCTGGTATCTGGGTAAAATGTTGCAAGCGATGGACGCATGCCGATGAACCTTGGCTTTCTTGGCAAGGGCACCCCCGCCCACCCCGACGCCTTGGTTAACAACTCGAACCCGGGGCTTGTGGCCTTAAACTGCCCCAAGAATGGGGGACCACAAA |
| G19 | AACTTCAGGCGTCGATCATCATCGGCGTGGCCACGGACATCATCGCGGGCGAGGGCAAGATCCTCACGGCCGGCGGCATCGACACCCACGTGCACTTCATCAGCCCGGATCAGATCGAGACGGCGCTGGTCTCCGGCATCACGACCATGATCGGCGGCGGGACGGGCCCGGGGGAGTCGACCAAGGCCACCACGATCACGCCCGGCGCGTGGAACATCCACGGCATGCTGCGCTCCTTCGAGCACTTCCCCATGAACTTCGGGCTGCTCGGCAAGGGCCACGGCTCCGCGATCCAGCCCATGGCGGAGCAGATCCGCGCCGGTGCGATCGGGCTGAAGATCCACGAGGACTGGGGGCGGCACAA |
| G21_white | GGGATGCAGGGTCGACGAGACTCGTCATCGGCCCCTCCACCGAGGTCATGGCCGGCAACGGCATGATCATGACCGCCGGCGCGATCGACACCCACGTCCACTACGTCACCCCCGACCAGCTGACCGTGGGCCTGAACGCGGGCGTGACCACCGTGATCGGCGGCGGCACCGGCCCGGCCGAGGGCTCGAAGGCGGCGCTCGCCACGCCCGGCGAGTGGTGGCTGCGGCGGATGTTCCAGGGCCTGGACCCGTGGCCCGTGAACTTCATGTTCCTCGGCCGCGGCAACGCCATGAACGACGAGGCGCTGCTGGAGCAGGTCCGCGCCGGGATGGGCGGGTTCAAGATCCACGAAGACTGGGGCGCCAAAA |
| GW3 | CCCGCCAGGTCGAGTGCTGATCGGCCCGGCACCGAGATCATCGCGGGCGAGGGCCGCATCCTGACCGCGGGCGCCATGGACGCGCATATCCATTTCATCTGCCCGCAGCAGATCGACGATGCGCTGCATTCGGGCATCACCACCATGCTGGGCGGCGGCACCGGCCCGGCGCATGGCACGCTGGCCACGACCTGCACCCCCGGCCCTTGGCATATCGGCCGCATGATGCAGGCCGCCGATGCCTTGCCGATGAACCTGGCCTTTGCCGGCAAGGGCAATGCCGCGCTGCCCGCCGCGCTGGAGGAACAGGTCCGCGCCGGGGCCTGCGCGCTGAAGCTGCACGAGGACTGGGGGCACAACA |
| P4 | ATTAAGTTATGGCTGGTCTCGCGCTTGGACGGGCACCGATGCATCTCCGGCGAGCACCTGATCCTCACGGCCGGCGGCATCGATGCGCACGTGCACTTCATCTCCCCGCAGCAGGCCGAGGCCGCGCTCAGCAATGGCGTGACTTCGCTGTTTGGCGGCGGCATCGGCCCCACCGACGGCACCAATGGCACGACCATCACGCCCGGCACGTGGAACGTGGAGATGATGCTCCGCTCGTTCGACGGCTGGCCGGTCAATGCAGGCGTTCTGGGCAAGGGCAACTGCTCGACCCGTCTGCCGATGGAAGAGCAGCTGCGCGCCGGCGTCATGGGCCTCAAGATCCACGAAGACTGGGGCACAACAA |
| P8.1 | CGAACGAAGTCGATCATCATCGGCCCGGCACTGAGGTGTCTCAGGCGAGGCGAGCATCCTCACGGTGGGCGGCTTTGAGCTCATATCCACTATATCTGCCCGCAACAGATCGAGGACGCGCTGATGACTGGCCTCACCACCATGCTGGGCGGAGGCACCGGGCCAGCCACTGGCTCTTTGGCCACCACCTGTACTCCCGGTGCCTGGCATCTTGGCCAAATGATGCAAGCCGCGGATGACTTGCCGATGAACCTGGGCCTGGTCGGCAAGGGAAATGCCTCTCTGCCGGAAGCGCTGGAAGAGCAGGTCGAGGCCGGCGCCTGCGCGCTCAAACTGCACGAAGACTGGGGGAGCAACAA |
| P12 | GCAACGGGCGTCGCTGATCTCGGGCCGGGCACCGAGATCATCGCGGGCGAGGGACCCATCCTGACGCCCGGCGGGTTCGACTGCCACATCCACTATATCTGTCCGCAGCAGGTCGAACACGCGCTGCATTCCGGCGTCACCACCCTGCTGGGCGGCGGCACCGGCCCTGCTCATGGCACGCTGGCCACCACCTGCACCCCCGGCCCGTGGCACATCCCCCGCATGCTGGAGGCCTGCGCCCAACTGCCCGTCAACTTCGGCATCGCTGGCAAGGGCAACGCCAGCCAGCCCGCCCCGCTGGAGGAACAGGTCCGCGCGGGTGCCTGCGCCCTGAAGCTGCACGAAGACTGGGGCACCACAAGAG |

**Supplementary Table 1-1.** The amino acid sequence of *ureC* gene of eight different UPB. G13: *Oceanospirillum linum*; G15: *Marinomonas pollencensis*; G19: *Staphylococcus saprophyticus*; G21_white: *Brachybacterium conglomeratum*; GW3: uncultured *Vibrio* sp.; P4: *Vibrio* sp.; P8.1: *Halomonas venusta*; P12: *Vibrio* sp..

| **Isolate** | **Amino acid sequence of *ureC* gene** |
| --- | --- |
| G13 | GDGVSADRPGTEIIAGEGRILTAGAMDAHIHFICPQQIDDALHSGITTMLGGGTGPAHGTLATTCTPGPWHIGRMMQAADALPMNLAFAGKGNAALPAALEEQVRAGACALKLHEDWGHN |
| G15 | HHVDSPLDRYRGHCRGGSILTAGGFDAHIHFICPQQIEEALHSGVTTWIGGGTGPAHGTLATTCTPGPWYLGKMLQAMDACR-TLAFLARAPPPTPTPWLTTRTRGLWP-TAPRMGDHK |
| G19 | LQASIIIGVATDIIAGEGKILTAGGIDTHVHFISPDQIETALVSGITTMIGGGTGPGESTKATTITPGAWNIHGMLRSFEHFPMNFGLLGKGHGSAIQPMAEQIRAGAIGLKIHEDWGRH |
| G21_white | DAGSTRLVIGPSTEVMAGNGMIMTAGAIDTHVHYVTPDQLTVGLNAGVTTVIGGGTGPAEGSKAALATPGEWWLRRMFQGLDPWPVNFMFLGRGNAMNDEALLEQVRAGMGGFKIHEDWGAK |
| GW3 | PARSSADRPGTEIIAGEGRILTAGAMDAHIHFICPQQIDDALHSGITTMLGGGTGPAHGTLATTCTPGPWHIGRMMQAADALPMNLAFAGKGNAALPAALEEQVRAGACALKLHEDWGHN |
| P4 | IKLWLVSRLDGHRCISGEHLILTAGGIDAHVHFISPQQAEAALSNGVTSLFGGGIGPTDGTNGTTITPGTWNVEMMLRSFDGWPVNAGVLGKGNCSTRLPMEEQLRAGVMGLKIHEDWGTT |
| P8.1 | RTKSIIIGPALRCLRRGEHPHGGRL-AHIHYICPQQIEDALMTGLTTMLGGGTGPATGSLATTCTPGAWHLGQMMQAADDLPMNLGLVGKGNASLPEALEEQVEAGACALKLHEDWGSN |
| P12 | ATGVADLGPGTEIIAGEGPILTPGGFDCHIHYICPQQVEHALHSGVTTLLGGGTGPAHGTLATTCTPGPWHIPRMLEACAQLPVNFGIAGKGNASQPAPLEEQVRAGACALKLHEDWGTTR |

**3.5. The urea consumption in medium**

**Supplementary Table 2.** One-way ANOVA and multiple comparison of urea content in medium between groups on 1 d, 2 d, and 3 d.

|  |  | **Time** | **df** | **F** | **Significance** |
| --- | --- | --- | --- | --- | --- |
| One-way ANOVA | Inter-group | 1 d | 2 | 267.188 | 0.000 |
|  |  | 2 d | 2 | 160.566 | 0.000 |
|  |  | 3 d | 2 | 139.352 | 0.000 |
|  |  | **Time** | **I group** | **J group** | **Significance** |
| Multiple comparison | LSD | 1 d | G1 | G2 | 1.000 |
|  |  |  |  | G3 | 0.000 |
|  |  |  | G2 | G1 | 1.000 |
|  |  |  |  | G3 | 0.000 |
|  |  |  | G3 | G1 | 0.000 |
|  |  |  |  | G2 | 0.000 |
|  |  | 2 d | G1 | G2 | 0.798 |
|  |  |  |  | G3 | 0.000 |
|  |  |  | G2 | G1 | 0.798 |
|  |  |  |  | G3 | 0.000 |
|  |  |  | G3 | G1 | 0.000 |
|  |  |  |  | G2 | 0.000 |
|  |  | 3 d | G1 | G2 | 0.000 |
|  |  |  |  | G3 | 0.001 |
|  |  |  | G2 | G1 | 0.000 |
|  |  |  |  | G3 | 0.000 |
|  |  |  | G3 | G1 | 0.001 |
|  |  |  |  | G2 | 0.000 |

**Supplementary Table 3.** One-way ANOVA and multiple comparison of uptake rate of urea between groups on 1 d, 2 d, and 3 d.

|  |  | **Time** | **df** | **F** | **Significance** |
| --- | --- | --- | --- | --- | --- |
| One-way ANOVA | Inter-group | 1 d | 2 | 267.188 | 0.000 |
|  |  | 2 d | 2 | 4.738 | 0.058 |
|  |  | 3 d | 2 | 34.123 | 0.001 |
|  |  | **Time** | **I group** | **J group** | **Significance** |
| Multiple comparison | LSD | 1 d | G1 | G2 | 1.000 |
|  |  |  |  | G3 | 0.000 |
|  |  |  | G2 | G1 | 1.000 |
|  |  |  |  | G3 | 0.000 |
|  |  |  | G3 | G1 | 0.000 |
|  |  |  |  | G2 | 0.000 |
|  |  | 2 d | G1 | G2 | 0.818 |
|  |  |  |  | G3 | 0.044 |
|  |  |  | G2 | G1 | 0.818 |
|  |  |  |  | G3 | 0.032 |
|  |  |  | G3 | G1 | 0.044 |
|  |  |  |  | G2 | 0.032 |
|  |  | 3 d | G1 | G2 | 0.004 |
|  |  |  |  | G3 | 0.000 |
|  |  |  | G2 | G1 | 0.004 |
|  |  |  |  | G3 | 0.011 |
|  |  |  | G3 | G1 | 0.000 |
|  |  |  |  | G2 | 0.011 |

**3.6. Physiological parameters of *G. lemaneiformis* cultured in different conditions**

**Supplementary Table 4.** One-way ANOVA and multiple comparison of the NH_4_^+^ content in *G. lemaneiformis* between groups on 1 d, 2 d, and 3 d.

|  |  | **Time** | **df** | **F** | **Significance** |
| --- | --- | --- | --- | --- | --- |
| One-way ANOVA | Inter-group | 1 d | 2 | 31.184 | 0.001 |
|  |  | 2 d | 2 | 27.429 | 0.001 |
|  |  | 3 d | 2 | 17.544 | 0.003 |
|  |  | **Time** | **I group** | **J group** | **Significance** |
| Multiple comparison | LSD | 1 d | G1 | G2 | 0.000 |
|  |  |  |  | G3 | 0.002 |
|  |  |  | G2 | G1 | 0.000 |
|  |  |  |  | G3 | 0.070 |
|  |  |  | G3 | G1 | 0.002 |
|  |  |  |  | G2 | 0.070 |
|  |  | 2 d | G1 | G2 | 0.002 |
|  |  |  |  | G3 | 0.074 |
|  |  |  | G2 | G1 | 0.002 |
|  |  |  |  | G3 | 0.000 |
|  |  |  | G3 | G1 | 0.074 |
|  |  |  |  | G2 | 0.000 |
|  |  | 3 d | G1 | G2 | 0.002 |
|  |  |  |  | G3 | 0.768 |
|  |  |  | G2 | G1 | 0.002 |
|  |  |  |  | G3 | 0.003 |
|  |  |  | G3 | G1 | 0.768 |
|  |  |  |  | G2 | 0.003 |

**Supplementary Table 5.** One-way ANOVA and multiple comparison of the urea content in *G. lemaneiformis* between groups on 1 d, 2 d, and 3 d.

|  |  | **Time** | **df** | **F** | **Significance** |
| --- | --- | --- | --- | --- | --- |
| One-way ANOVA | Inter-group | 1 d | 2 | 0.979 | 0.428 |
|  |  | 2 d | 2 | 13.985 | 0.006 |
|  |  | 3 d | 2 | 11.461 | 0.009 |
|  |  | **Time** | **I group** | **J group** | **Significance** |
| Multiple comparison | LSD | 1 d | G1 | G2 | 0.525 |
|  |  |  |  | G3 | 0.496 |
|  |  |  | G2 | G1 | 0.525 |
|  |  |  |  | G3 | 0.211 |
|  |  |  | G3 | G1 | 0.496 |
|  |  |  |  | G2 | 0.211 |
|  |  | 2 d | G1 | G2 | 0.002 |
|  |  |  |  | G3 | 0.053 |
|  |  |  | G2 | G1 | 0.002 |
|  |  |  |  | G3 | 0.028 |
|  |  |  | G3 | G1 | 0.053 |
|  |  |  |  | G2 | 0.028 |
|  |  | 3 d | G1 | G2 | 0.064 |
|  |  |  |  | G3 | 0.046 |
|  |  |  | G2 | G1 | 0.064 |
|  |  |  |  | G3 | 0.003 |
|  |  |  | G3 | G1 | 0.046 |
|  |  |  |  | G2 | 0.003 |

**Supplementary Table 6.** One-way ANOVA and multiple comparison of the total cellular nitrogen in *G. lemaneiformis* between groups on 1 d, 2 d, and 3 d.

|  |  | **Time** | **df** | **F** | **Significance** |
| --- | --- | --- | --- | --- | --- |
| One-way ANOVA | Inter-group | 1 d | 2 | 8.288 | 0.019 |
|  |  | 2 d | 2 | 6.343 | 0.033 |
|  |  | 3 d | 2 | 18.339 | 0.003 |
|  |  | **Time** | **I group** | **J group** | **Significance** |
| Multiple comparison | LSD | 1 d | G1 | G2 | 0.043 |
|  |  |  |  | G3 | 0.196 |
|  |  |  | G2 | G1 | 0.043 |
|  |  |  |  | G3 | 0.007 |
|  |  |  | G3 | G1 | 0.196 |
|  |  |  |  | G2 | 0.007 |
|  |  | 2 d | G1 | G2 | 0.115 |
|  |  |  |  | G3 | 0.136 |
|  |  |  | G2 | G1 | 0.115 |
|  |  |  |  | G3 | 0.012 |
|  |  |  | G3 | G1 | 0.136 |
|  |  |  |  | G2 | 0.012 |
|  |  | 3 d | G1 | G2 | 0.007 |
|  |  |  |  | G3 | 0.097 |
|  |  |  | G2 | G1 | 0.007 |
|  |  |  |  | G3 | 0.001 |
|  |  |  | G3 | G1 | 0.097 |
|  |  |  |  | G2 | 0.001 |

**3.7. Stable isotopic analysis of δ^15^N in *G. lemaneiformis***

**Supplementary Table 7.** One-way ANOVA and multiple comparison of the δ^15^N accumulation in *G. lemaneiformis* between groups on 1 d, 2 d, and 3 d.

|  |  | **Time** | **df** | **F** | **Significance** |
| --- | --- | --- | --- | --- | --- |
| One-way ANOVA | Inter-group | 1 d | 2 | 81.133 | 0.000 |
|  |  | 2 d | 2 | 165.904 | 0.000 |
|  |  | 3 d | 2 | 23.431 | 0.001 |
|  |  | **Time** | **I group** | **J group** | **Significance** |
| Multiple comparison | LSD | 1 d | G1 | G2 | 0.470 |
|  |  |  |  | G3 | 0.000 |
|  |  |  | G2 | G1 | 0.470 |
|  |  |  |  | G3 | 0.000 |
|  |  |  | G3 | G1 | 0.000 |
|  |  |  |  | G2 | 0.000 |
|  |  | 2 d | G1 | G2 | 0.046 |
|  |  |  |  | G3 | 0.000 |
|  |  |  | G2 | G1 | 0.046 |
|  |  |  |  | G3 | 0.000 |
|  |  |  | G3 | G1 | 0.000 |
|  |  |  |  | G2 | 0.000 |
|  |  | 3 d | G1 | G2 | 0.049 |
|  |  |  |  | G3 | 0.005 |
|  |  |  | G2 | G1 | 0.049 |
|  |  |  |  | G3 | 0.001 |
|  |  |  | G3 | G1 | 0.005 |
|  |  |  |  | G2 | 0.001 |

**Supplementary Table 8.** Repeated measures ANOVA of the δ^15^N accumulation in *G. lemaneiformis* between groups during the whole culture period.

|  | Ⅲ-type quadratic sum | df | Mean square | F | Significance |
| --- | --- | --- | --- | --- | --- |
| Intercept | 2554092709.252 | 1 | 2554092709.252 | 1080.867 | .000 |
| Group | 554634173.101 | 2 | 277317086.551 | 117.358 | .000 |
| Error | 14178018.792 | 6 | 2363003.132 |  |  |
